# An Infrared Spectroscopic Investigation of Nitric Oxide Binding on Isolated Cobalt Cluster Cations

**DOI:** 10.1021/acs.jpca.5c02939

**Published:** 2025-06-24

**Authors:** Peter T. Rubli, Christian T. Haakansson, Philip A. J. Pearcy, Ruby G. Spratt, Joost M. Bakker, Peter D. Watson, Stuart R. Mackenzie

**Affiliations:** † Department of Chemistry, Chemistry Research Laboratory, University of Oxford, Mansfield Road, Oxford OX1 3TA U.K.; ‡ HFML-FELIX, Toernooiveld 7, Nijmegen 6525 ED, the Netherlands; § Institute for Molecules and Materials, Radboud University, Heyendaalseweg 135, Nijmegen 6525 AJ, the Netherlands

## Abstract

Nitric oxide binding to and/or dissociation on isolated cobalt cluster cations, Co*_n_* ^+^ (*n* = 3–14), has been investigated using a combination of infrared multiple photon dissociation spectroscopy and density functional theory. Rich vibrational structure in the 300–800 cm^–1^ spectral region reflects predominantly dissociative adsorption, though a minor molecularly bound isomer cannot be ruled out. Inert messenger tagging reveals nitrogen and oxygen atoms bound in bridged and/or three-atom sites. The calculated potential energy surface associated with the reaction between NO and Co_3_^+^ confirms only submerged barriers to dissociation and unusual full insertion of the N atom, which binds to all three metal atoms. The second NO adsorbed also dissociates on all clusters studied here, with the smallest cluster, [Co_3_N_2_O_2_]^+^-Ar*_m_*, adopting an unusual planar cyclic structure with O atoms and an N_2_ molecule inserted between adjacent Co atoms.

## Graphical abstract

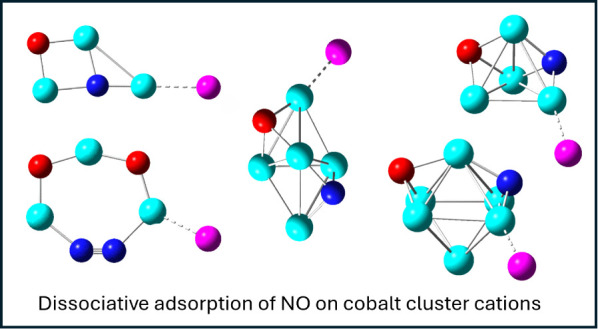

## 1 Introduction

The importance of nitrogen oxides (NO*_x_*) and their negative effects on both the environment and human health is widely acknowledged.^1^ These gaseous pollutants play a central role in harmful environmental phenomena such as acid rain and smog, and contribute to the degradation of stratospheric ozone.^2–4^ In addition to being produced naturally, nitric oxide is a known byproduct of internal combustion engines and its catalytic reduction has attracted considerable interest.^5^ In turn, this has led to strict emissions policies including the requirement for three-way catalytic converters in exhaust systems,^6^ the chemistry of which is based on highly dispersed transition metals.

The role of defects as key active sites at which heterogeneous catalytic processes involving transition metals occur has long been recognized.^7,8^ Small gas-phase transition metal clusters provide tractable model systems for some active sites providing valuable insight into the basic chemistry without the complexity associated with solvents or substrates.^9,10^ Information on fundamental cluster-ligand interactions can reveal the nature of the initial binding process, the reaction products formed, and any cluster size and charge effects.^11^ Infrared multiple photon dissociation (IRMPD) spectroscopy has emerged as a powerful technique particularly well-suited to this task.^12–14^ Harnessing the sensitivity of mass spectrometry for infrared spectroscopy, IRMPD allows the structure of size-selected metal clusters and their interactions with small molecules such as H_2_, CO, H_2_O, CO_2_, CH_4_ and NH_3_ to be investigated in exquisite detail.^11,15^

The reactivity of transition metal clusters with nitrogen oxides has been studied extensively both in single collision reactivity and spectroscopically. Nitrous oxide binding to clusters such as Rh*_n_* ^±^,^16–20^ Au*_n_* ^+^,^21,22^ Co*_n_* ^+^,^21^ and Pt*_n_* +^23^ has been studied by IRMPD as has N_2_O as a ligand in metal–ligand complexes.^24–29^ Conventional reactivity studies of NO with a range of transition metal clusters including Nb*_n_* ^+^,^30,31^ Ni*_n_* ^±^,^32–34^ Co*_n_* ^+^,^35–40^ and Rh*_n_* ^±^,^41,42^ have been reported leading to a recent review of the catalytic reduction of NO on metal clusters.^43^ In several recent cases, IRMPD has been used to characterize the nature of nitrosyl adsorption including on Au*_n_* ^±^,^44,45^ Ir*_n_* ^+^,^46^ and, especially, Rh*_n_* ^+^.^47–49^ NO has also been studied as a ligand in gas-phase metal ion-nitrosyl complexes, M^+^(NO)*_n_* (*M* = Fe,^50^ Cu,^51^ Ag,^52^ Au,^52^ and group 9 atoms^53^).

Relevant to the present work, cobalt is much cheaper and more abundant^54,55^ than the platinum group metals which form the active component in automobile catalytic converters.^56^ It has an additional advantage in studies based on mass spectrometric detection in that it is monoisotopic. Co*_n_* ^+^ clusters thus provide attractive systems for the investigation of reactivity with NO. IRMPD spectroscopy using a free electron laser was used to study the structures of small Co*_n_* ^+^ (*n* = 4–8) clusters and found that the Ar atoms used as “inert” tags significantly distorted the structures of the smallest clusters.^57^

The open-shell nature of NO presents challenges in quantum chemical calculations of metal nitrosyl clusters. Geometries and frequencies have been calculated for NO binding to first-row transition metal atoms^58^ and NO binding to small Co*_n_* and Co*_n_* ^+^ clusters has been explored using density functional theory (DFT).^59–61^ For cluster sizes larger than Co_2_^+^, nitric oxide is predominantly dissociatively adsorbed. Experimentally, NO reactivity toward Co*_n_* ^+^ has been investigated using guided ion beam studies and ion cyclotron resonance mass spectrometry.^35–40^ Measurement of absolute cross sections indicate that dissociative chemisorption is dominant, with the cross section rising sharply at *n* = 4.^37,38^ Sequential additions lead to decomposition of NO and loss of N_2_ resulting in formation of [Co*_n_* O_2_]^+^ and [Co*_n_* O_2_(NO)]^+^ clusters.^35,40^

In this work, we present an infrared photodissociation study of NO binding to Co*_n_* ^+^ clusters, with structural assignment aided by comparison with structures calculated at the density functional theory level. IRMPD has been employed in conjunction with argon-tagging to study [Co*_n_* (NO)*_y_*]^+^ (*n* = 3–14, *y* = 1,2) clusters using tunable IR light from the Free Electron Laser for IntraCavity Experiments (FELICE) at the HFML-FELIX facility. Spectra focused on the 300–830 cm^–1^ range, chosen to identify metal-O, metal-N and metal-NO modes and thereby determine the nature of the chemisorption.

## 2 Experimental and Computational Methods

All experiments were performed using the molecular beam apparatus coupled to the FELICE beamline which has been described in detail previously.^62,63^ Cobalt clusters were produced by pulsed laser ablation of a rotating and translating cobalt rod using the second harmonic of a Nd:YAG laser (∼20 mJ, 20 Hz). Ablation takes place in a growth channel (3 mm diameter, 60 mm long) where clustering is initiated by 3-body collisions with a 3% argon in helium carrier gas mixture admitted via a pulsed valve (General valve, Series 9).

Following cluster formation, the gas pulse passes through a copper reaction channel (3 mm diameter, 45 mm long) at which point NO is injected via a second pulsed valve. The reaction channel was cooled to −40 °C and is fitted with a converging-diverging nozzle (∼0.7 mm diameter) through which the gas pulse expands into the vacuum. The resulting cluster beam is irradiated by the FELICE (300–830 cm^–1^) infrared beam within the extraction region of a reflectron time-of-flight (ReTOF) mass spectrometer into which ions are extracted and hit a microchannel plate detector. Control over the IR fluence to which the molecular beam is exposed, is achieved by adjusting the relative overlap of the FELICE and molecular beams. A compromise between maximum (depletion) signal and spectral saturation is reached by translating the entire molecular beam instrument relative to the infrared focus. The fluence of FELICE can be tuned by translating the molecular beam experiment along the laser beam in or out of the focus. For this work, FELICE macropulse fluences ranged from 1 to 9 J/cm^2^. Argon atoms are used as weakly bound messenger tags, which are lost following infrared absorption, providing a mass spectrometric signature of IR absorption

1 $$[{\mathrm{Co}}_{n}{\mathrm{NO}]}^{+}{\mathrm{‐Ar}}_{m}+h\nu \to [{\mathrm{Co}}_{n}{\mathrm{NO}]}^{+}{\mathrm{‐Ar}}_{m-x}+x\mathrm{Ar}$$

Figure 1a shows a representative time-of-flight mass spectrum produced in the ablation source. The mass spectrum shows significant Co*_n_* ^+^ and [Co*_n_* NO]^+^ clusters up to *n* ∼ 25 (see Figure S1 for extended mass spectrum). Here we adopt the convention of using square brackets, [Co*_n_* NO]^+^, for species whose molecular structures cannot be determined from their mass alone. Although the mass spectrum is highly congested, almost all species can be unambiguously assigned (see Figure 1b). In addition to [Co*_n_* NO]^+^-Ar*_m_* and [Co*_n_* N_2_O_2_]^+^-Ar*_m_*, other peaks in the mass spectrum such as the [Co*_n_* O_2_]^+^ adducts are observed upon addition of NO. This suggests substantial chemistry occurs on the Co*_n_* ^+^ cluster surfaces within the source region, similar to that observed by Anderson et al., under single collision conditions in which adsorption of multiple NO molecules leads to the decomposition of NO and production of [Co*_n_* O_2_]^+^.^35^ The same study also observed reaction-induced fragmentation (Co atom loss), with the degree of fragmentation decreasing for larger clusters and this cannot be excluded here either, although the (much) higher pressure conditions of the cluster source probably help stabilize clusters.

**Figure 1:**
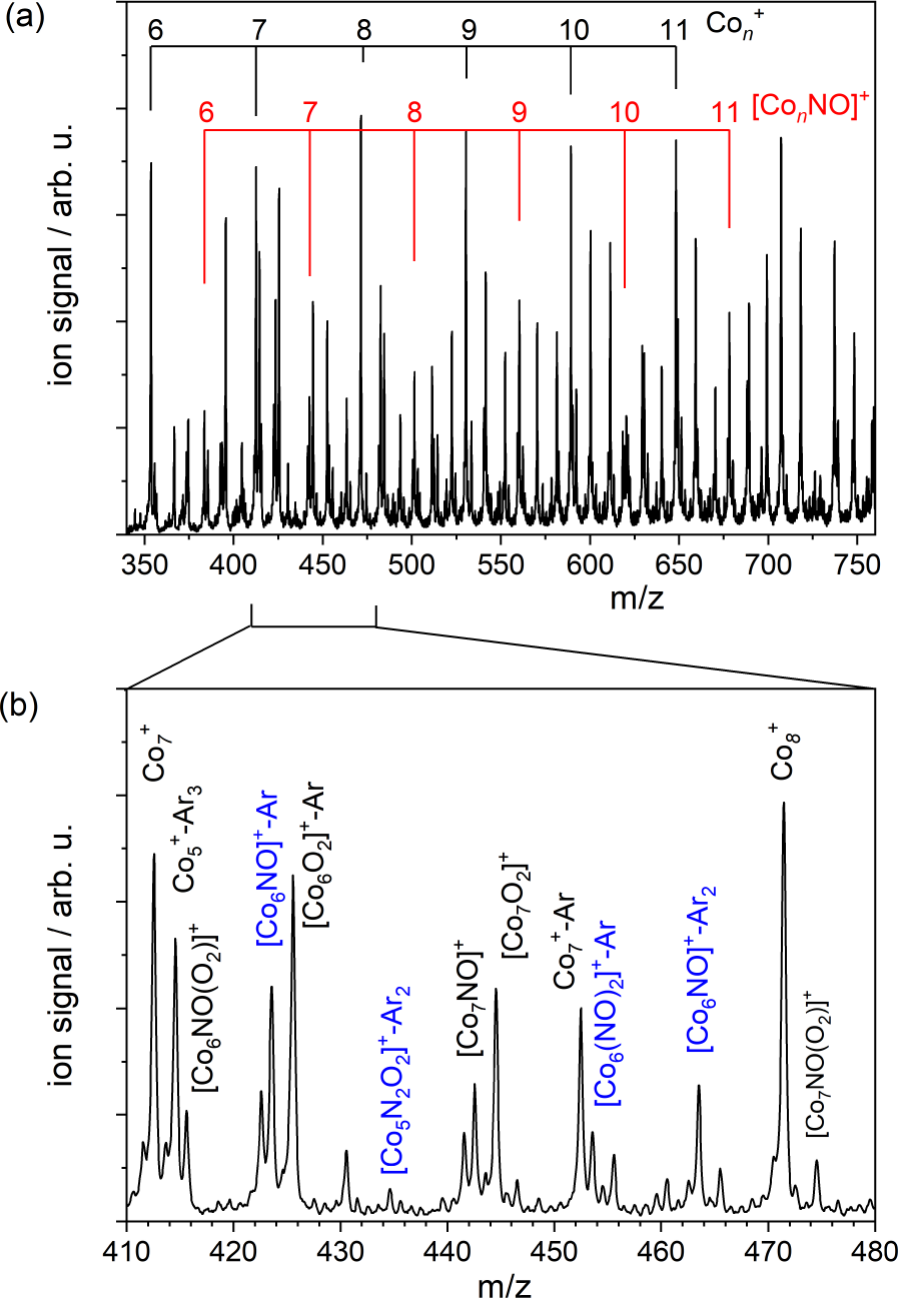
Time-of-flight mass spectrum produced by ablation of a cobalt rod in the presence of a 3% Ar in He carrier gas mix with NO introduced via a second nozzle. Panel (a) shows the extensive range of Co*_n_* ^+^ and [Co*_n_* NO]^+^ clusters formed in this work. Panel (b) shows a magnified view of the 410 – 480 *m*/*z* region in which other species including [Co*_n_* N_2_O_2_]^+^, [Co*_n_* O_2_]^+^, and Ar-tagged complexes are observed.

To allow for corrections of long-term source fluctuations, the experiment operates at 20 Hz with FELICE at 10 Hz, enabling acquisition of reference mass spectra between FELICE macropulses. In the absence of mass selectivity, IRMPD spectra of all species are recorded simultaneously. An important consequence of this is that larger clusters can fragment into small clusters e.g., Ar loss from [Co_3_NO]^+^-Ar_2_ results in enhancement on the [Co_3_NO]^+^-Ar mass channel. Depending on the relative ion signals of different species, this can lead to unwelcome artifacts in the IRMPD spectra such as simultaneous production and depletion of the same species. To account for this, and shot to shot fluctuations in the cluster efficiency, we report spectra of IRMPD yields, *Y*(*v̅*) as a function of excitation wavenumber based on branching ratios. For FELICE on spectra, we calculate the branching ratio, *B*(*v̅*) of the ion intensities, *I*, of [Co*_n_* NO]^+^-Ar*_m_* clusters for a specific *n*:

2 $$B\left(\mathrm{\nu ̅}\right)=\frac{\overset{m_{\max}}{\underset{m=k}{\sum }}I_{{[{\mathrm{Co}}_{n}\mathrm{NO}]}^{+}{\mathrm{‐Ar}}_{m}}}{\overset{m_{\max}}{\underset{m=0}{\sum }}I_{{[{\mathrm{Co}}_{n}\mathrm{NO}]}^{+}{\mathrm{‐Ar}}_{m}}}$$

Here, *m*_*max*_ is the maximum number of Ar tags observed in the mass spectrum for [Co*_n_* NO]^+^-Ar*_m_* of a particular *n*. *k* is the smallest value of *m* in [Co*_n_* NO]^+^-Ar*_m_* for which only Ar loss is observed under irradiation. Hence, the numerator in *B*(*v̅*)­is the sum of the mass spectrometric intensities of clusters exhibiting only depletion, while the denominator is the total sum of all the intensities for a particular *n*. The value of *k* varies between clusters of different *n* value: *k* = 4 for *n* = 3–5, and *k* = 1 for *n* = 6–14 (the latter binding many fewer Ar atoms in general). It must be noted that due to a mass degeneracy of [Co*_n_* NO]^+^-Ar_3_ and [Co*_n_*_+2_O_2_]^+^, clusters of *m* = 3 are not included in this branching ratio.

Once *B*(*v̅*) has been determined, the IRMPD yield, *Y*(*v̅*) is calculated as the logarithm of the fractional depletion using

3 $$Y\left(\mathrm{\nu ̅}\right)=-\frac{1}{P\left(\mathrm{\nu ̅}\right)}\ln\left[\frac{B\left(\mathrm{\nu ̅}\right)}{B_{0}}\right]$$

where *P*(*v̅*)­is the macropulse energy which accounts for the change in laser power over the wavelength range and *B*_0_ is the branching ratio when FELICE is off. [Co*_n_* N_2_O_2_]^+^-Ar*_m_* spectra are generated using the same methodology. To determine the intracavity pulse energy, a small fraction of the light from the FELICE cavity is directed onto a power meter. Full macropulse energies were typically in the range 200–700 mJ for these experiments. Wavenumber calibration is performed by directing FELICE output onto a grating spectrometer. The spectral bandwidth was is approximately ∼0.4% fwhm of the central frequency.

To assist in the assignment of spectra, predicted geometries, relative energies, and simulated IR spectra of relevant (i.e., energetically low-lying) isomers have been calculated using DFT with the Gaussian 16 electronic structure software package^64^ using the B3P86 ^65,66^ /Def2TZVP^67,68^ functional/basis set combination. This level of theory was chosen as it has been shown previously to reproduce very well the IRMPD spectra of similar cobalt clusters.^69^ In addition to chemical intuition, a modified Kick3 algorithm was used to generate an array of novel potential structures to adequately sample the conformer space of any given cluster.^70,71^ Calculated IR spectra are convoluted with Lorentzian line profiles (fwhm = 8 cm^–1^) to aid comparison with experimental spectra. The calculated IR spectra are presented here without frequency scaling. To map a potential reaction pathway, the synchronous transit-guided quasi-Newton method was employed to optimize transition state structures and determine activation barriers.^72^ Intrinsic reaction coordinate (IRC) calculations were used to confirm that transition states were in fact saddle points between minima.^73^ Binding energies (B.E.) of Ar tag atoms are obtained by calculating the difference between the parent cluster energy and the sum of the daughter fragment energies.

## 3 Results and Discussion

### 3.1 Overview of [Co*_n_* NO]^+^-Ar*_m_* Infrared Spectra

Figure 2 shows an overview of the IRMPD spectra of the [Co*_n_* NO]^+^ clusters (*n* = 3–14) recorded in the depletion/enhancement channels of the corresponding [Co*_n_* NO]^+^-Ar*_m_* clusters as outlined above. Although some spectral features are common to different clusters, the spectra vary considerably with cluster size, both in the number of vibrational bands observed (which ranges from ca. 5 to 10 in the cluster size range shown here) and in their respective linewidths. Some clusters exhibit broad absorptions such as the band centered at 610 cm^–1^ for *n* = 5 with a fwhm ≈ 100 cm^–1^, while others, such as the *n* = 6 spectrum, have much sharper line widths of *ca*. 10 cm^–1^.

**Figure 2:**
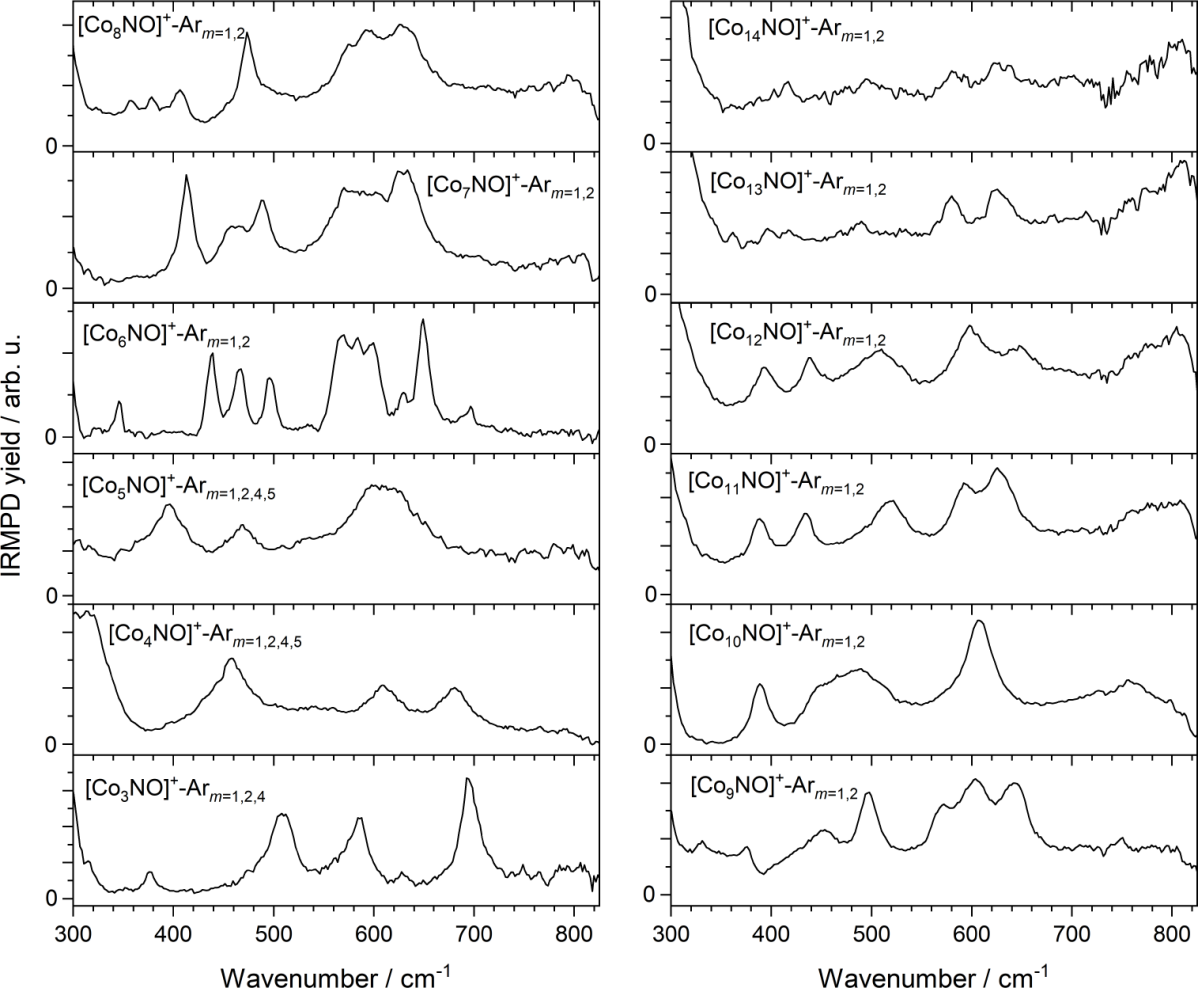
IRMPD spectra of [Co*_n_* NO]^+^-Ar*_m_* (*n* = 3–14, *m* = 1–5) species recorded in the depletion of the indicated Ar-tagged species. For these clusters, *k* = 4 for *n* = 3–5, and *k* = 1 for *n* = 6–14.

Several factors can contribute to broad line widths in FEL IRMPD spectra. First, the Ar binding on which the IRMPD technique depends, is relatively strong to [Co*_n_* NO]^+^ clusters, particularly for *n* = 3 (binding energy ∼0.3 eV), 4 (∼0.6 eV), and 5 (∼0.2 eV) (Figures 3, S3b and S4b respectively). For photon energies in the 300–800 cm^–1^ region (0.037–0.1 eV), even allowing for the internal energy of the clusters, multiple photon absorption is required to drive Ar loss in most cases. Interestingly, the unusually sharp line widths observed in the spectrum of the *n* = 6 cluster, reflect particularly weak Ar binding to this cluster (<0.01 eV, see Figure S5b). The binding strength of the Ar tag atoms will be discussed further in the subsequent sections. Second, Ar atoms can also bind in different sites on a given cluster giving rise to isomers with different spectra. Moreover, they likely experience wide amplitude motion in each local minimum and possibly even hopping between minima. Finally, under typical conditions employed, many clusters bind multiple Ar atoms and the spectra shown reflect multiple species (see Figure 2). When a [Co*_n_* NO]^+^-Ar*_m_* cluster undergoes absorption induced fragmentation, it is likely that the [Co*_n_* NO]^+^-Ar*_m_*_–1_ daughter is produced with significant internal excitation, potentially driving isomerization or Ar migration.

**Figure 3:**
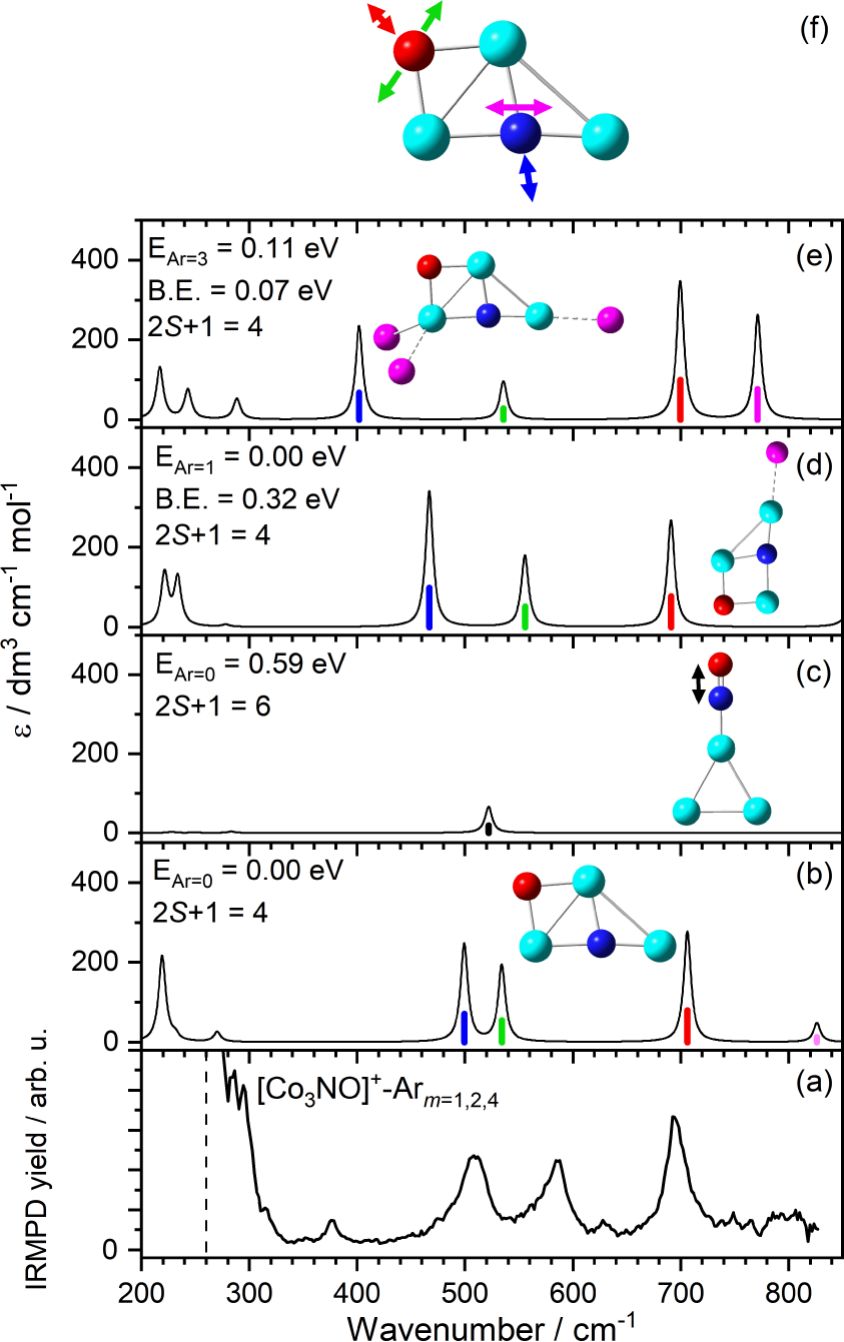
Comparison of (a) the experimental IRMPD spectrum of [Co_3_NO]^+^-Ar_m_ (*m* = 1, 2, 4) and (b)–(e) the simulated spectra of low-energy structures of [Co_3_NO]^+^-Ar*_m_* (*m* = 0, 1, 3) calculated at the B3P86/Def2TZVP level of theory. The energies of the structures (E_Ar=*m*_) are given relative to the lowest energy isomer. Also shown are the calculated binding energies (B.E.) associated with loss of one argon atom and spin multiplicities of the calculated structures. Panel (f) shows the color-coded mode vectors associated with the calculated vibrations in [Co_3_NO]^+^.

As the size of the cluster increases, additional factors lead to further broadening and absorption across nearly the entire wavelength range scanned. The number of possible overtone and/or combination bands increases rapidly with the number of normal modes as does the vibrational internal energy; E_vib_ ∼ 0.28 eV for [Co_3_NO]^+^ to 0.47 eV for [Co_6_NO]^+^ at the 230 K nozzle temperature. The high intracavity pulse energies of FELICE can drive these weaker transitions.

The apparent onset of a band at the edge of our scanning region 300 cm^–1^ is observed in many of the spectra. These bands are real, appearing before power correction (Figure S2) and are assigned to some of the higher wavenumber Co–Co modes based on simulated spectra for the structures assigned to the three smallest [Co*_n_* NO]^+^ clusters (*n* = 3–5), see Figure 3 below and Figure S2a.

### 3.2 [Co_3_NO]^+^ Spectrum and Simulations

In an attempt to begin to assign the vibrational spectra we start with the smallest cluster for which we could record a spectrum. Figure 3 shows the comparison between the experimental IRMPD spectrum of [Co_3_NO]^+^-Ar*_m_* and simulated IR spectra for calculated energetically low-lying structures of [Co_3_NO]^+^-Ar*_m_*_=0,1,3_. Under the backing pressure conditions employed here, the smallest clusters (*n* = 3–5) bind up to 5 argon atoms and simultaneous depletion and enhancement in individual mass channels is a particular problem (see Figure S6a–c for depletion/enhancement spectra recorded in each [Co*_n_* NO]^+^-Ar*_m_* (*n* = 3–5, *m* = 0–5) channel). For this reason, Figure 3a shows the IRMPD yield spectrum as outlined in eqs 2 and 3.

The experimental spectrum comprises strong, well-resolved absorption bands at 508, 585, and 695 cm^–1^, together with weaker features at 376 and 628 cm^–1^. These are sufficient to deduce important features of the nitrosyl binding motif including that the NO is dissociatively adsorbed on the cluster. The calculated spectrum for the molecularly–bound isomer, shown in Figure 3c, exhibits only a single weak cluster–ligand intermolecular stretch in this region, at 522 cm^–1^. We cannot rule out the presence of this band in the experimental spectrum but it is clear that this isomer cannot account for the full spectrum. We recorded survey scans in the mid-IR for [Co*_n_* NO]^+^ (*n* = 3–14) without Ar tagging in the 630–2000 cm^–1^ spectral range where we would expect strong NO absorption^50,53,74^ (Figure S13) but no peaks characteristic of a molecularly bound NO were observed. We thus conclude that the molecularly bound isomer is, at most, a minor component.

Figure 3b shows the simulated spectrum generated for the putative global minimum energy structure of [Co_3_NO]^+^, a quartet state with dissociatively bound NO which is calculated to lie 0.59 eV lower in energy than the molecularly bound isomer. This spectrum shows much better agreement with three major peaks in the experimental spectrum. This structure features O–atom binding in a bridged (Co–O–Co) site with the N atom fully inserting into the trigonal Co_3_^+^ to bind to all three Co atoms. Despite the lack of scaling factor applied, the calculated vibrations at 706, 534, and 499 cm^–1^ are in reasonable agreement with the observed bands which thus are assigned to the symmetric oxide stretch, the antisymmetric oxide stretch (or in-plane wag), and symmetric nitride stretch, respectively as shown in Figure 3f. The two symmetric stretches are in exceptional agreement with the experimental spectrum while the calculated O atom wagging motion (or antisymmetric stretch) is calculated to lie 50 cm^–1^ to the red of the experimental band.

This is a further example, similar pure Co*_n_* ^+^,^57^ in which the presence of Ar tag atoms significantly perturbs the vibrational spectrum as illustrated in Figure 3d, e, and Ar tagging may provide a plausible explanation for the weaker bands in the spectrum. Figure S6a shows spectra for each individual [Co_3_NO]^+^-Ar*_m_* species. Note that these spectra are measured as depletion of the [Co_3_NO]^+^-Ar*_m_* channel or enhancement in intensity of the corresponding [Co_3_NO]^+^-Ar*_m_*_–1_ mass channel. Although many characteristics are preserved between the spectra, such as the strong 700 cm^–1^ absorption band addition of successive Ar tags moves other vibrational bands. This is particularly true at the red end of this spectrum (below 400 cm^–1^) where multiple Ar binding leads to a significant red-shift in the nitride vibrations. These changes accompany structural rearrangement in which the planar symmetry of [Co_3_NO]^+^ is broken by the terminal Co atom in [Co_3_NO]^+^-Ar*_m_* moving out of plane, leading to a 170° Co–N–Co bond angle.

### 3.3 Potential Energy Pathway for NO Adsorption on Co_3_^+^

The IR spectrum of [Co_3_NO]^+^ (Figure 3) clearly indicates dissociative NO adsorption. In order to explore this and N atom insertion into the Co_3_ structure, we have calculated the reactive potential energy surface in Figure 4. The putative global minimum (GM) lies 2.52 eV below the separated Co_3_^+^ + NO asymptote with only submerged barriers between them. The lowest molecularly bound structure (I1) represents an entrance–channel complex, rearrangement from which leads to NO binding across a Co–Co bond (I2). The first major barrier encountered (TS2, *ca*. 1 eV) sees N insertion into the Co–Co bond and formation of the Co–O–Co bridge bond. This significantly weakens the N–O bond which lengthens from 1.20 Å to 1.35 Å ultimately breaking at the highest transition state (TS3) before the N atom settles into its three (Co-) atom binding site. Even TS3 lies 0.33 eV below the asymptote, indicating a plausible reaction pathway leading directly to the global minimum structure despite the significant structural rearrangement involved.

**Figure 4:**
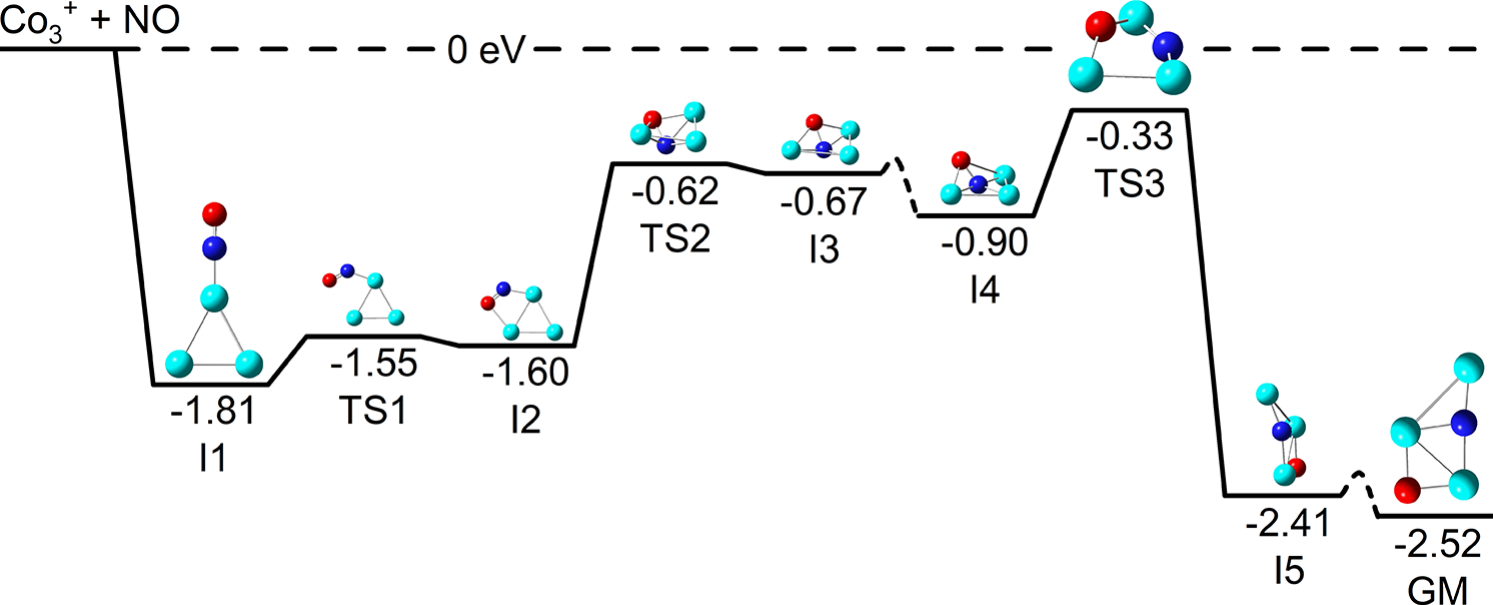
Reaction pathway for the dissociation of nitric oxide on the Co_3_^+^ cluster (2*S* + 1 = 4) surface calculated at the B3P86/Def2TZVP level of theory. Only submerged barriers are encountered on the journey from Co_3_^+^ + NO to the global minimum structure. Barriers shown as dashed lines indicate minimal structural rearrangements between the adjacent intermediate structures, investigation of which is beyond the scope of this work.

The experimental evidence suggests insertion of the N atom into the base Co*_n_* ^+^ structure occurs only in the *n* = 3 case (Figures 5, S3, S4 and S6). One explanation for this can be found in the work of Hales et al. which reports bond dissociation energies of bare cobalt clusters, Co*_n_* ^+^ (*n* = 2–18).^75^ In this size range, Co_3_^+^ has the lowest bond dissociation energy (*D*(Co*_n_*_–1_^+^–Co) = 2.04 ± 0.13 eV) reflecting its 2-dimensional nature, with the equivalent Co loss energy for *n* ≥ 5 exceeding 2.8 eV. Although insertion involves only internal bond breaking rather than atom loss, it is likely that a similar trend is followed. It is also notable that Anderson et al. observed Co atom loss in reactions of NO + Co*_n_* ^+^ (*n* < 13) under mass–selective single collision conditions.^35^ By contrast, Koyama et al. observed no significant Co*_n_* ^+^ dissociation for the same reaction in a gas cell filled with He, suggesting collisional stabilization following the initial NO adsorption.^40^ In our experiments here, NO binds in the relatively high pressure environment of the cluster channel. Hence, we do not believe our clusters suffer significant Co atom loss, but we cannot prove it.

**Figure 5:**
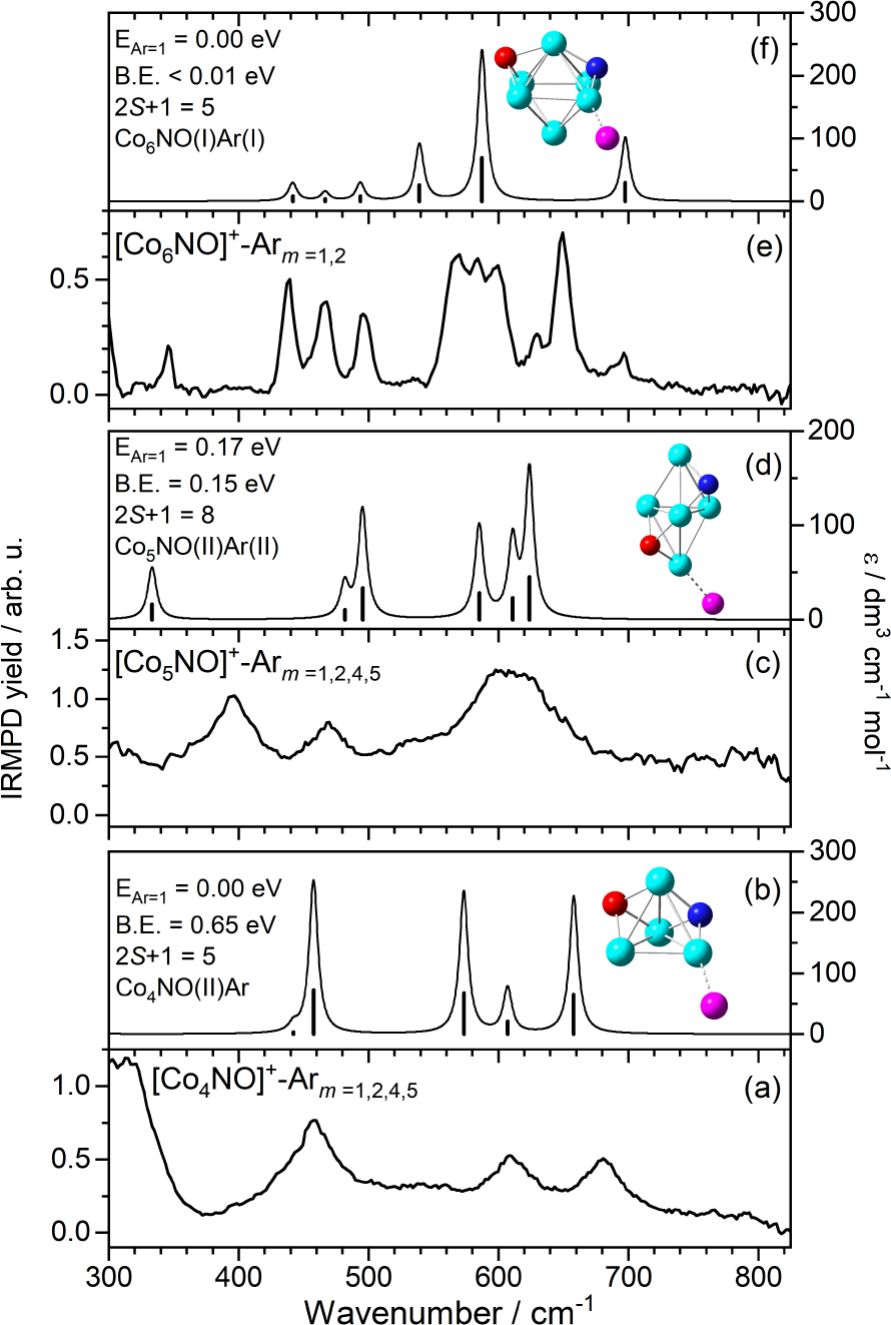
Experimental IRMPD spectra of (a) [Co_4_NO]^+^-Ar*_m=1,2,4,5_* (c) [Co_5_NO]^+^-Ar*_m=1,2,4,5_* and (e) [Co_6_NO]^+^-Ar*_m=1,2_* are compared with the harmonic spectra of low-lying isomers for [Co*_n_* NO]^+^-Ar (*n* = 4–6) shown in (b), (d), and (f), respectively. Structures are calculated at the B3P86/Def2TZVP level of theory and their energies (E_Ar=1_) are given relative to the energy of the lowest energy isomer calculated for the same number of Ar tags. Calculated binding energies (B.E.) for the Ar tags and spin multiplicities are also shown.

### 3.4 NO Adsorption on Larger Co*_n_* ^+^ Clusters (*n* = 4, 5, 6)

Figure 5 shows comparisons between the experimental and simulated IRMPD spectra of [Co*_n_* NO]^+^-Ar*_m_* (*n* = 4–6). Comprehensive comparisons between experimental and calculated spectra for multiple calculated isomers which support these assignments can be found in Figures S3–S5. The experimental spectrum of [Co_4_NO]^+^–Ar*_m_* shows three clear but broad bands at 456, 609, and 681 cm^–1^, with a potential additional band toward 300 cm^–1^. The simulated spectrum for structure Co_4_NO­(II)Ar (*i.e*., the second lowest energy isomer of Co_4_NO^+^, tagged with an Ar atom, Figure 5b) is in reasonable agreement with the main features. This structure exhibits N and O adsorbed to the tetrahedral Co_4_^+^ cluster in three atom binding sites, consistent with the global minimum structure found by Facio-Muñoz et al.^60^ This allows assignment of the antisymmetric N stretch, the in-phase symmetric N and O stretches, and combined in- and out-of-phase antisymmetric stretches of N and O to the bands at 681, 609, and 456 cm^–1^ respectively. The broad absorption spanning the range 515–575 cm^–1^ is tentatively assigned to the out-of-phase antisymmetric stretches of N and O (calculated at 573 cm^–1^). This cluster exhibits anomalously high Ar binding energy of 0.65 eV which may contribute to the broad spectrum.

The spectrum of [Co_5_NO]^+^-Ar*_m_* (Figure 5c) exhibits three broad peaks in the 300–700 cm^–1^ range centered at 395, 468, and 610 cm^–1^. The calculated spectrum for Co_5_NO­(II)­Ar­(II) (Figure 5d with Ar bound atop the axial Co atom) accounts satisfactorily for the experimental spectrum. This structure is similar to that of *n* = 4 in that NO dissociates upon binding which generates O and N atoms bound to three atom sites on the trigonal bipyramidal Co_5_^+^. The bands at 468 and 610 cm^–1^ are attributed to groups of vibrational modes consisting of a mixture of symmetric and antisymmetric oxide and nitride stretches (Figure S4c). This assignment supports the observed broadness of the bands due to the probable convolution of these modes. The third peak observed at 395 cm^–1^ could either be the antisymmetric oxide stretch calculated at 333 cm^–1^, or an NO rocking motion of the molecularly bound Co_5_NO­(IV) structure (Figure S4a) calculated at 393 cm^–1^.

The experimental spectrum of [Co_6_NO]^+^-Ar*_m_* (Figure 5e) is qualitatively different to all others studied in this size range, with many more, well-resolved bands. The spectrum calculated for the putative global minimum [Co_6_NO]^+^-Ar structure (Co_6_NO­(I)­Ar­(I)) agrees reasonably well with the experimental spectrum, particularly for the three bands observed at 438, 466, and 495 cm^–1^ which are assigned to oxide and nitride stretches (Figure S5c). Additionally, the band at 696 cm^–1^ is assigned to the asymmetric N stretch at 698 cm^–1^. The two bands observed at 650 and 630 cm^–1^ are attributed to the same motion, in isomers with alternative Ar binding positions (660 and 623 cm^–1^ for isomers Co_6_NO­(I)­Ar­(II) and Co_6_NO­(I)­Ar­(III), respectively, see Figure S5b). The blended symmetric oxide stretches in the same three isomers also account for the broader feature spanning ca. 550 to 600 cm^–1^ (Figure S5b). The underlying Co_6_NO structure remains firmly octagonal/tetragonal bipyramidal with N and O atoms bound in three atom binding sites. Such a mixture of isomers is consistent with the maximum observed depletion of ∼30% each for the three bands around 550–600 cm^–1^ (see Figure S6d).

In all cases for *n* ≥ 4, the calculated [Co*_n_* NO]^+^ molecularly bound isomers have weak modes which lie close to features observed experimentally, typically in the region 550–575 cm^–1^. While we cannot discount the presence of these bands, it is clear that molecularly bound structures do not account for all the spectral features observed experimentally. Perhaps understandably given the increasing numbers of accessible electronic (spin) states and isomers, the level of overall agreement of experimental spectra with individual simulated spectra get markedly worse as the cluster size increases. This is not helped by the fact that no scaling has been applied to the harmonic frequencies calculated (as which scaling to use is unclear). Finally, the spectral effects of Ar tagging appear more pronounced in the region of vibrations involving the O- and *N*- atoms than further to the red in the Co–Co modes.

### 3.5 Multiple NO Adsorption on Co*_n_* ^+^ Clusters

Under conditions of slightly higher nitric oxide partial pressure, it was possible to observe binding of multiple NO molecules to all the clusters observed in this study. The IRMPD spectra of the [Co*_n_* N_2_O_2_]^+^-Ar*_m_* clusters (*n* = 3–14, *m* = 0–4) are shown in Figure 6 and all exhibit (broad) vibrational bands in this region of the spectrum. One immediate observation is that the differences between spectra are less significant than for the [Co*_n_* NO]^+^ clusters with persistent bands present in the spectra of several clusters, e.g., around 610 cm^–1^ for all clusters *n* ≥ 6.

**Figure 6:**
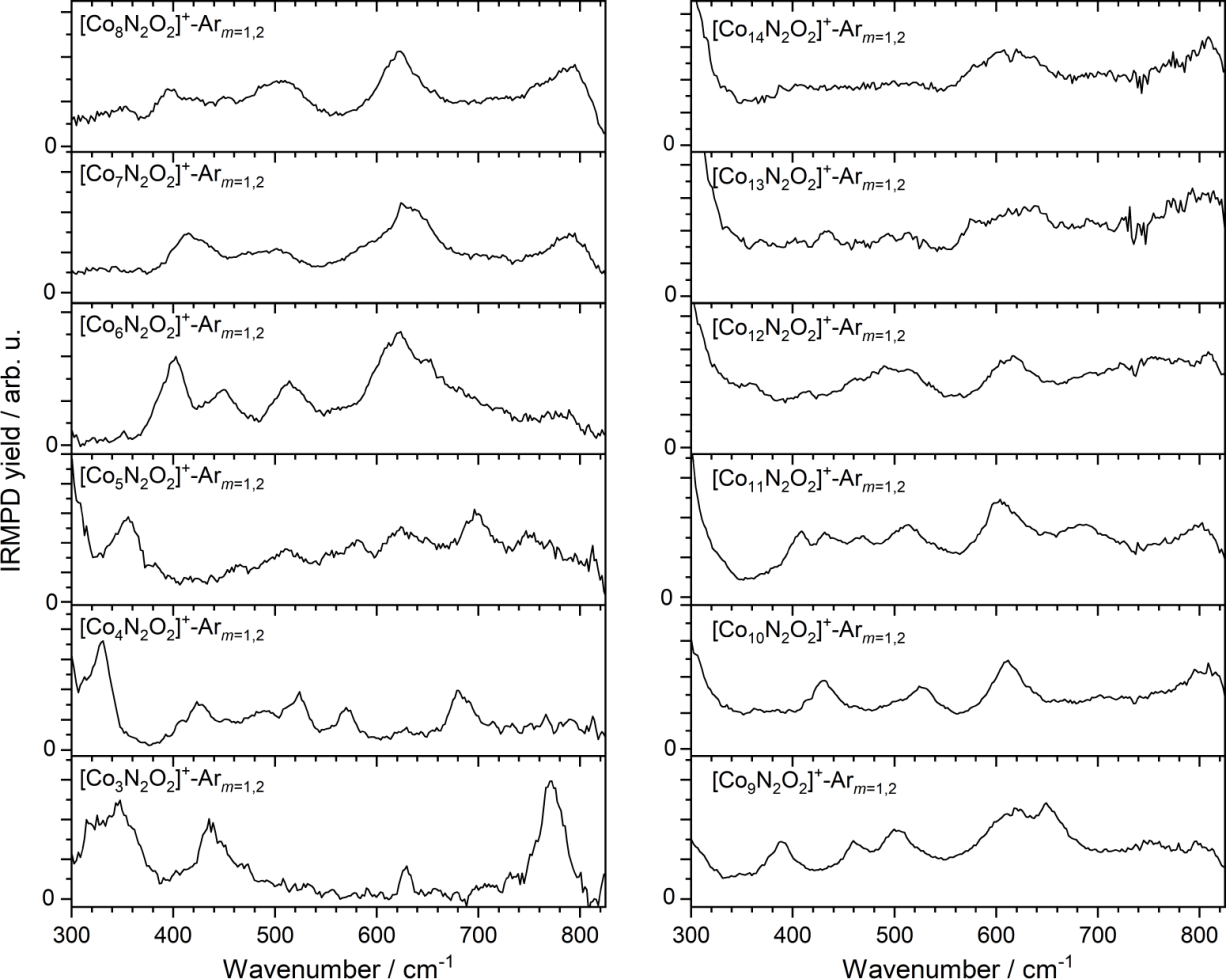
IRMPD spectra of [Co*_n_* N_2_O_2_]^+^-Ar*_m_* (*n* = 3–14, *m* = 1, 2) species recorded in the depletion of the indicated Ar-tagged species. For these clusters, *k* = 2 for *n* = 3 and 4, and *k* = 1 for *n* ≥ 5.

IR bands above 700 cm^–1^ are more prominent for the [Co*_n_* N_2_O_2_]^+^ clusters, especially the intense feature in the [Co_3_N_2_O_2_]^+^ spectrum at 775 cm^–1^. Figure 7 shows the comparison between experimental spectrum and that calculated for the global minimum structure for this cluster. All the energetically low-lying structures adopt intriguing cyclic structures with no Co–Co bonds. The global minimum structure exhibits two adjacent bridged Co–O–Co moieties, and a Co–N=N–Co structure with vibrational assignments given in Figure S11. Both the bare structure and its Ar-tagged analogue agree reasonably well with experimental spectrum, and suggests the band at 770 cm^–1^ arises from an intense in-phase dioxide stretching mode (Figure S11b). There is also evidence of the antisymmetric O atom stretch and out-of-phase dioxide stretching modes (calculated at 691 and 634 cm^–1^). Importantly, the bands observed at 347 and 435 cm^–1^ provide evidence for modes associated with in-plane N=N rocking and wagging modes. As convincing as this structural assignment is, it would imply extensive chemistry on the surface of the cluster following dissociative adsorption of two nitric oxide molecules. The exothermicity involved in formation of the N=N bond might suggest that the chemistry took place on a larger cluster which cooled by Co atom evaporation consistent with the single collision reactivity studies.^35^ Certainly N_2_ loss is commonly observed in adsorption of multiple NO molecules of transition metal clusters, including cobalt.^35,41–43^

**Figure 7:**
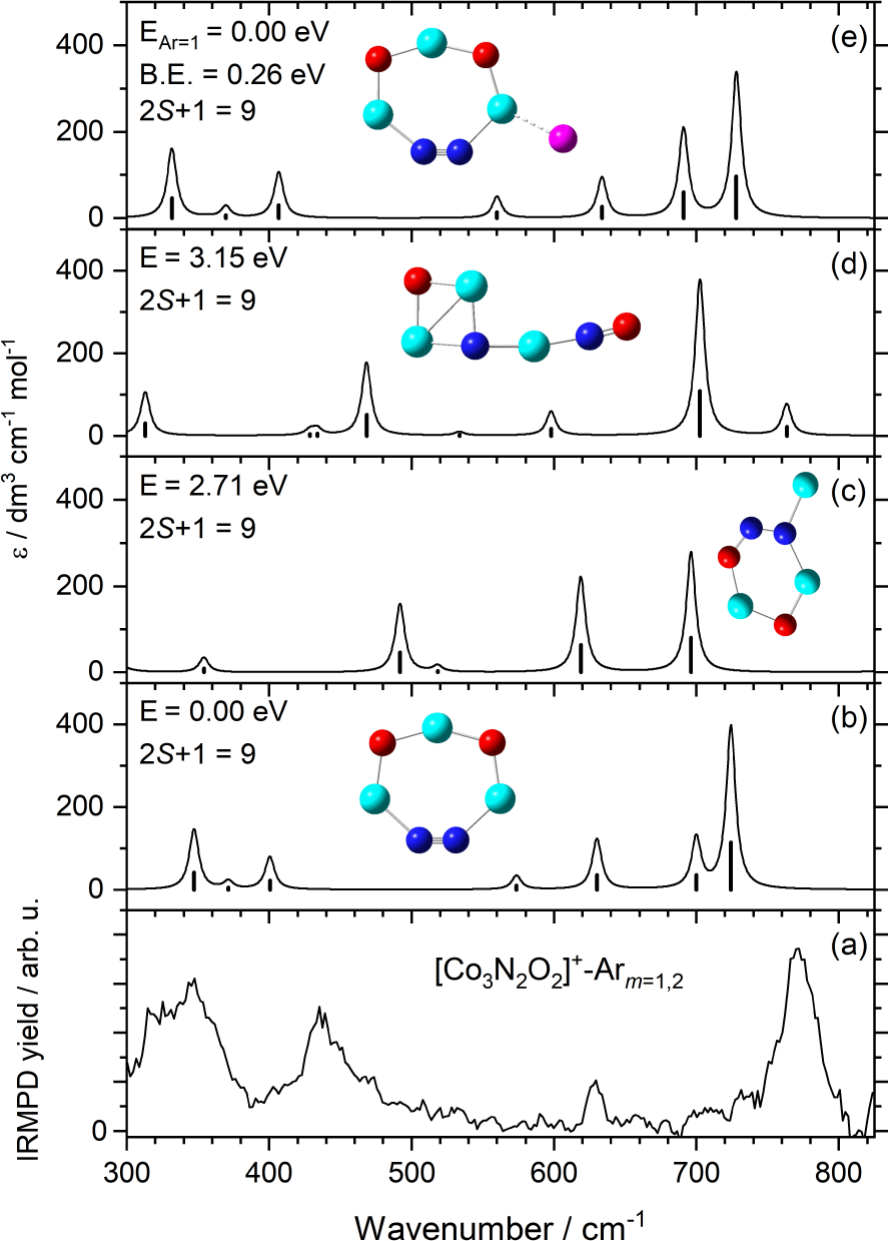
Comparison between (a) the experimental IRPD spectrum of [Co_3_N_2_O_2_]^+^-Ar*_m_* (*m* = 1,2) and (b)–(e) the harmonic spectra of low-energy isomeric structures of [Co_3_N_2_O_2_]^+^-Ar*_m_* (*m* = 0,1) calculated at the B3P86/Def2TZVP level of theory. The energies of the structures (E_Ar=*m*_) are relative to the energy of the lowest energy isomer calculated for the same number of Ar tags. Calculated binding energies (B.E.) for the Ar tag and spin multiplicities are also shown.

The near planar ring structure appears unique to [Co_3_N_2_O_2_]^+^. Equivalent comparisons between the experimental and simulated spectra for [Co_4_N_2_O_2_]^+^-Ar*_m_* and [Co_5_N_2_O_2_]^+^-Ar*_m_* are shown in Figure S12 and indicate more familiar three-dimensional structures.

## 4 Summary and Conclusions

IRMPD spectroscopy employing the FELICE free electron laser has been used to study the dissociative chemisorption of nitric oxide on small isolated cobalt cluster cations. Spectra of [Co*_n_* NO]^+^-Ar*_m_* clusters (*n* = 3–14, *m* = 0–5) were recorded in the 300–800 cm^–1^ region, and all show rich and varied vibrational structure in the region of interest. Cluster structures have been assigned as far as possible by comparison with simulated spectra of calculated low-lying isomers and confirm the dominant dissociative nature of the adsorption. In this respect nitric oxide adsorption on Co*_n_* ^+^ is similar to NO binding to iridium doped rhodium clusters, Rh*_n_* Ir^+^.^76^ By contrast, molecular binding dominates NO adsorption at Au*_n_* +^44^ and Au_4_^–^ ^45^ while evidence for both molecular and dissociative adsorption is observed on pure Rh*_n_* +^43^ and Ir*_n_* +^46^ clusters.

The influence of Ar tags on the spectra of small cobalt clusters has been observed previously,^57^ and is shown here to lead to significant red-shifts for some vibrational modes. Transition state calculations for NO adsorption on Co_3_^+^ provide a plausible reaction pathway formation of the global minimum dissociatively bound isomer which involves insertion of the N atom into the base Co_3_^+^ structure.

All clusters were also found to bind a second NO molecule, and IRMPD spectra and simulations for these [Co*_n_* N_2_O_2_]^+^-Ar*_m_* clusters (*n* = 3–14, *m* = 0–4) are also presented which are consistent with dissociative binding for the second NO. The smallest cluster size, [Co_3_N_2_O_2_]^+^, adopts an interesting cyclic structure in which O atoms and an N_2_ moiety insert between Co atoms.

## Supplementary Material



## Notes

Additional time-of-flight mass spectra; details of macropulse power correction; comparison of experimental and simulated spectra for more cluster sizes (*n* = 4–6); effects of multiple Ar-tagging on IRMPD spectra; mass resolved fragmentation mass spectra confirming assignments (PDF)

## #

Western Australian School of Mines: Minerals, Energy and Chemical Engineering, Curtin University, Perth, Australia, 6102
