# Supplementary material for: An Infrared Spectroscopic Investigation of Nitric Oxide Binding on Isolated Cobalt Cluster Cations
Source: J Phys Chem A. 2025 Jun 24;129(26):5810–9. doi: 10.1021/acs.jpca.5c02939 (PMC12235619; doi:10.1021/acs.jpca.5c02939)
Supplement: Supplementary file 1 [file jp5c02939_si_001.pdf]

An Infrared Spectroscopic Investigation of Nitric Oxide Binding on Isolated Cobalt Cluster Cations

Peter T. Rubli,[a] Christian T. Haakansson,[a] Philip A. J. Pearcy,[a] Ruby G. Spratt,[a] Joost M. Bakker,[b] Peter D. Watson,[a†] and Stuart R. Mackenzie[a]*

[a] Department of Chemistry, University of Oxford, Chemistry Research Laboratory, Mansfield Road, Oxford, UK, OX1 3TA.

[b] Radboud University, Institute for Molecules and Materials, HFML-FELIX, Toernooiveld, 6525 ED Nijmegen, Netherlands

† Present address: Western Australian School of Mines: Minerals, Energy and Chemical Engineering, Curtin University, Perth, Australia, 6102

* Corresponding Author: stuart.Mackenzie@chem.ox.ac.uk

Supporting Information

The supporting information presented here comprises of results of DFT calculations performed on $[Co_nNO]^+$-Ar_m and $[Co_nN_2O_2]^+$-Ar_m clusters calculated at the B3P86/Def2TZVP level of theory. Included are optimized structures, simulated spectra and mode vectors, energies, cartesian coordinates, and harmonic frequencies. In addition, further analysis of mass spectral data is shown to demonstrate the variations in $[Co_nNO]^+$-Ar_m branching ratios between smaller ($n \leq 5$) and larger clusters.

Contents:

List of Figures

Figure S1: Time-of-flight mass spectrum of Co_n^+ and $[Co_nNO]^+$ clusters (n = 6–24)…………………4

Figure S2: Effect of power correction on IRMPD spectra due to FELICE intracavity power…………5

Figure S3: Calculated vibrational and experimental spectra of $[Co_4NO]^+$-Ar_m clusters……….……..6

Figure S4: Calculated vibrational and experimental spectra of $[Co_5NO]^+$-Ar_m clusters……….……..7

Figure S5: Calculated vibrational and experimental spectra of $[Co_6NO]^+$-Ar_m clusters……….……..8

Figure S6: Effect of successive Ar tagging on IRMPD spectra of $[Co_nNO]^+$ clusters (n = 3–6)..……9

Figure S7: Mass difference spectra recorded for $[Co_3NO]^+$-Ar_m clusters taken both on-resonance and off-resonance to confirm spectral assignment of these clusters……………………..10

Figure S8: Mass difference spectra recorded for $[Co_4NO]^+$-Ar_m clusters taken both on-resonance and off-resonance to confirm spectral assignment of these clusters……………………..11

Figure S9: Mass difference spectra recorded for $[Co_5NO]^+$-Ar_m clusters taken both on-resonance and off-resonance to confirm spectral assignment of these clusters……………………..12

Figure S10: Mass spectra illustrating the branching ratios of $[Co_nNO]^+$-Ar_m clusters (n = 3–6)……13

Figure S11: Calculated vibrational and experimental spectra of $[Co_3N_2O_2]^+$-Ar_m clusters…………14

Figure S12: Calculated vibrational and experimental spectra of $[Co_4N_2O_2]^+$-Ar_m and $[Co_5N_2O_2]^+$-Ar_m clusters……………………………………………………………………………..15

Figure S13: IRMPD spectra of $[Co_nNO]^+$ clusters (n = 3-14) in the 630-2000 cm^{-1} region………....16

Figure S14: IRMPD spectra of $[Co_nN_2O_2]^+$ clusters (n = 3-14) in the 630-2000 cm^{-1} region……….17

List of Tables

Table S1: Cartesian coordinates of optimized $[Co_3NO]^+$-Ar_m (m = 0, 1, 3) structures…...…………18

Table S2: Cartesian coordinates of the $[Co_3NO]^+$ intermediate isomers and transition state structures involved with the dissociative reaction pathway……………………..………19

Table S3: Cartesian coordinates of optimized $[Co_nNO]^+$-Ar (n = 4, 5, 6) structures………………...20

Table S4: Cartesian coordinates of optimized $[Co_3N_2O_2]^+$-Ar_m (m = 0, 1) structures………..…..21

Table S5: Calculated harmonic frequencies of $[Co_3NO]^+$-Ar_m (m = 0, 1, 3) structures…………….22

Table S6: Calculated harmonic frequencies of the $[Co_3NO]^+$ intermediate isomers and transition state structures involved with the dissociative reaction pathway……………………..23-24

Table S7: Calculated harmonic frequencies of $[Co_nNO]^+$-Ar (n = 4, 5, 6) structures………………..25

Table S8: Calculated harmonic frequencies of $[Co_3N_2O_2]^+$-Ar_m (m = 0, 1) structures…………..26-27

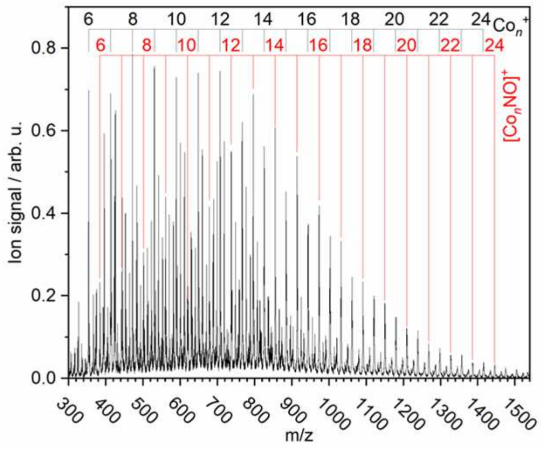

Figure S1: Time-of-flight mass spectrum produced by ablating a Co rod target in the presence of a 3% Ar in He carrier gas mix with pure NO introduced via a second nozzle. This expanded mass spectrum indicates the large range of Co_n^+ and $[Co_nNO]^+$ cluster sizes generated outside of those discussed in the main text.

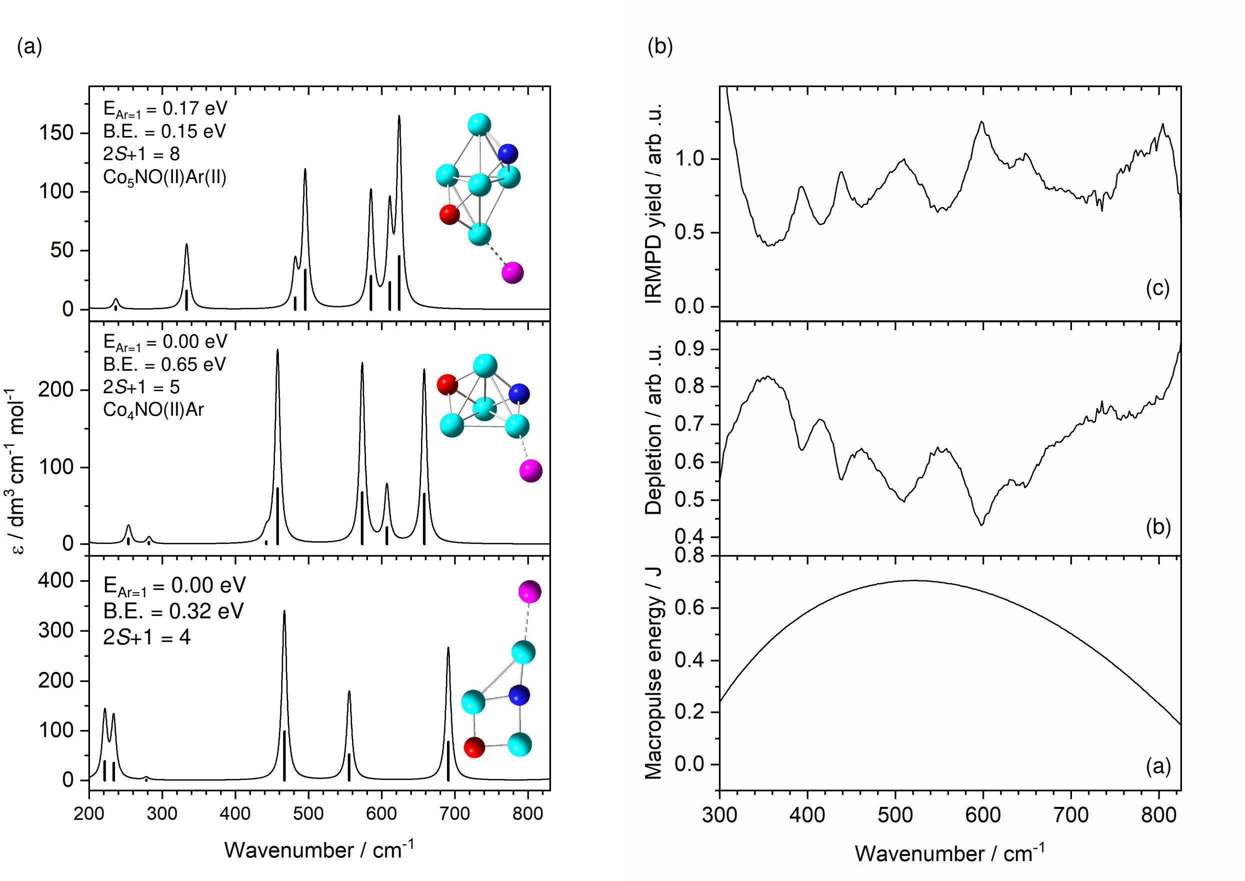

Figure S2: (a) Harmonic spectra for the three smallest $[Co_nNO]^+$-Ar cluster sizes calculated at the B3P86/Def2TZVP level of theory. The wavelength range is extended below 300 cm^{-1} to 200 cm^{-1} to illustrate the predicted presence of vibrational modes in that region. (b) The intra-cavity macropulse energy of FELICE (panel a) shown alongside the pure depletion (panel b, without power correction) and IRMPD yield (panel c, with power correction using equation 3 spectra for $[Co_{12}NO]^+$-Ar_m to illustrate the effect of power correction. For clarification, panel b shows $B(v)/B_0$ while panel c shows $Y(v)$ as in equation 3.

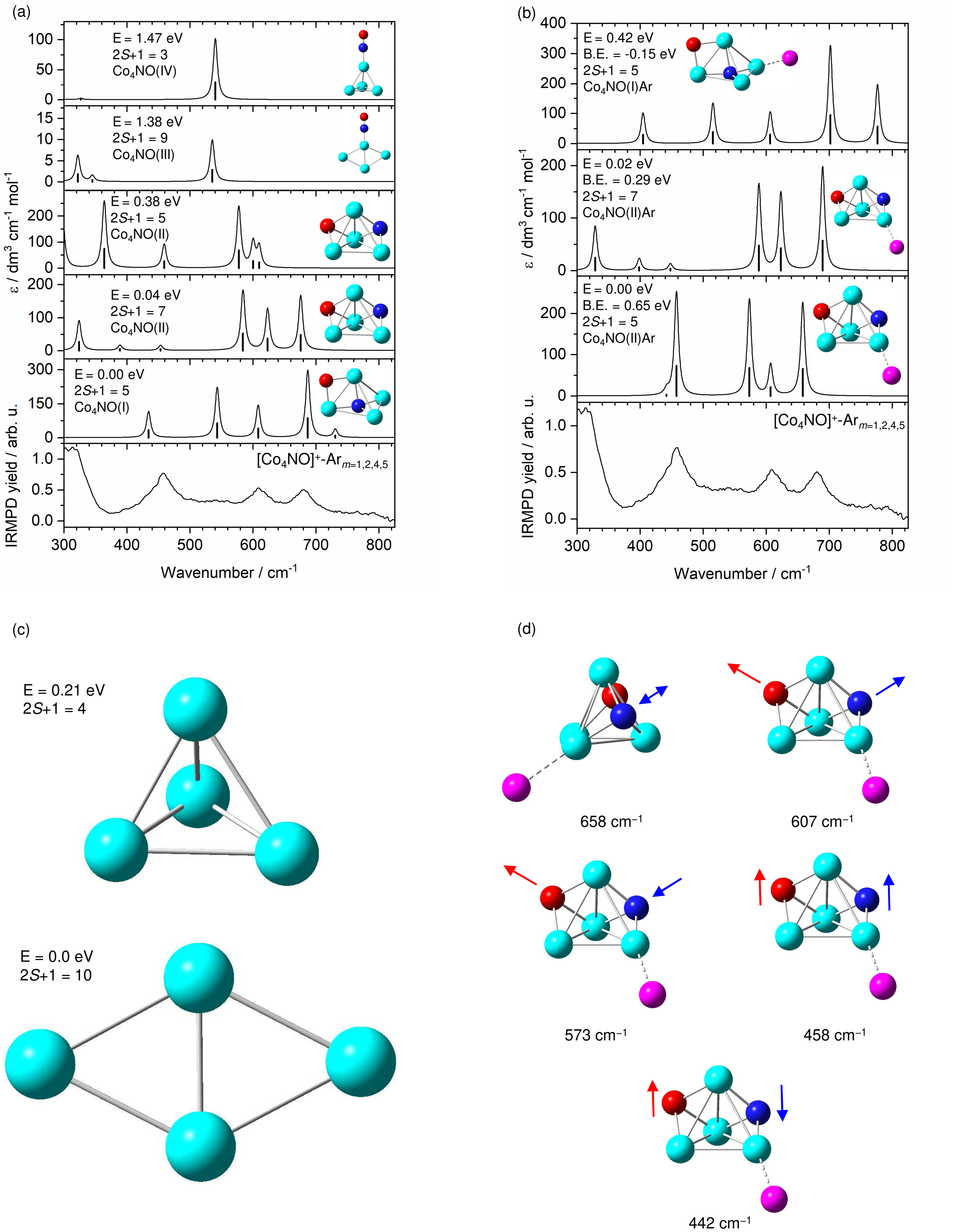

Figure S3: (a) The experimental IRMPD spectrum for $[Co_4NO]^+$-Ar_m compared to spectra calculated for low-lying isomeric forms of $[Co_4NO]^+$ with both molecular and dissociative NO binding motifs. (b) The experimental IRMPD spectrum for $[Co_4NO]^+$-Ar_m compared to spectra calculated for low-lying isomeric forms of $[Co_4NO]^+$-Ar. (c) The lowest energy simulated structures found for the planar rhombic and tetrahedral Co_4^+ isomers. (d) The frequencies and motions of vibrational modes calculated for the putatively assigned $[Co_4NO]^+$-Ar structure, Co_4NO(II)Ar with $2S+1 = 5$. Structures are calculated at the B3P86/Def2TZVP level of theory with relative energies (E), spin multiplicities, and binding energies (B.E.) for the Ar tags are also shown.

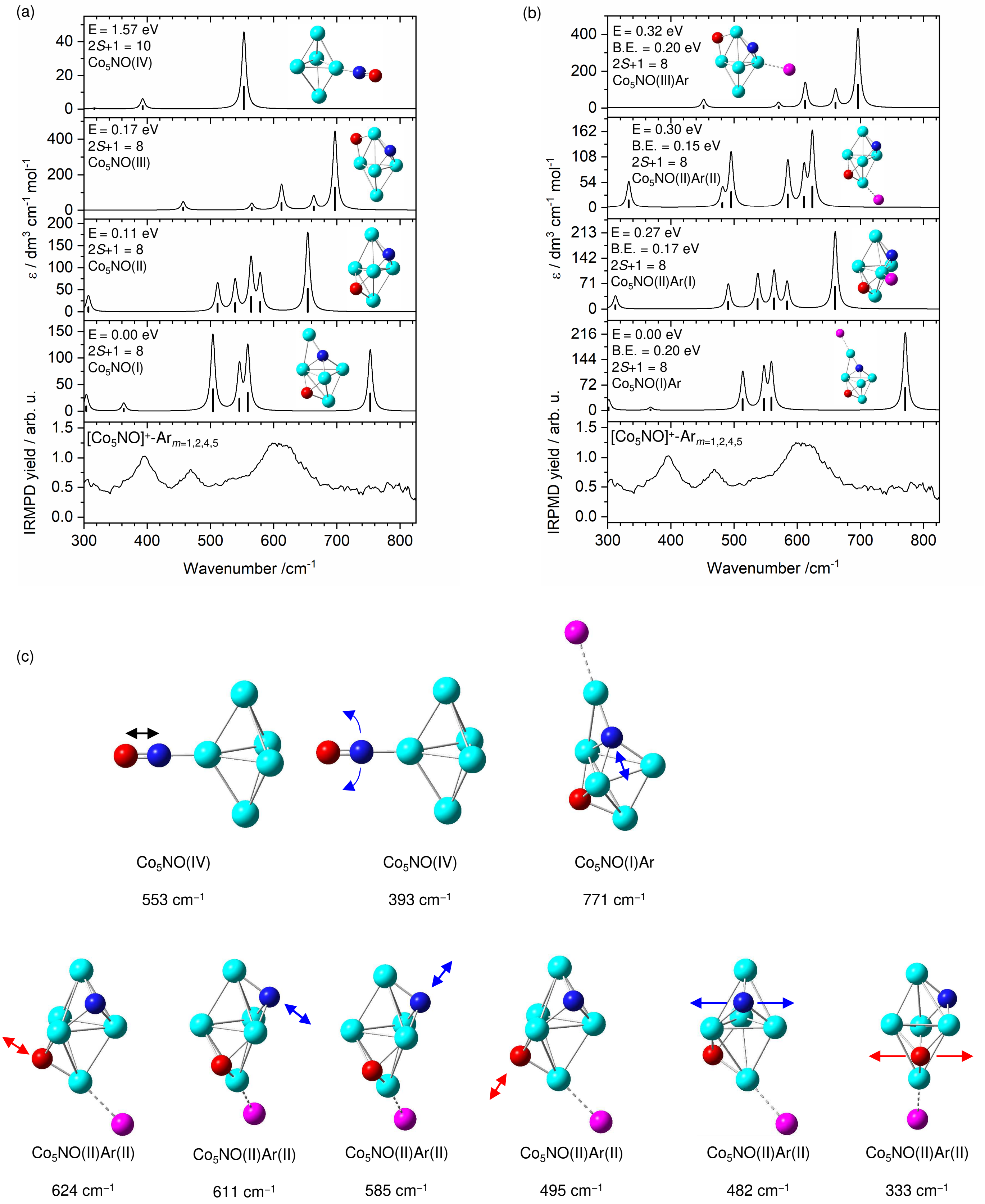

Figure S4: (a) The experimental IRMPD spectrum for $[Co_5NO]^+$-Ar_m compared to spectra calculated for low-lying isomeric forms of $[Co_5NO]^+$ with both molecular and dissociative NO binding motifs. (b) The experimental IRMPD spectrum for $[Co_5NO]^+$-Ar_m compared to spectra calculated for low-lying isomeric forms of $[Co_5NO]^+$-Ar. (c) The frequencies and motions of vibrational modes calculated for the molecularly bound Co_5NO(**IV**), Co_5NO(**I**)Ar, and putatively assigned Co_5NO(**II**)Ar(**II**) isomers. Structures are calculated at the B3P86/Def2TZVP level of theory with relative energies (E), spin multiplicities, and binding energies (B.E.) for the Ar tags are also shown.

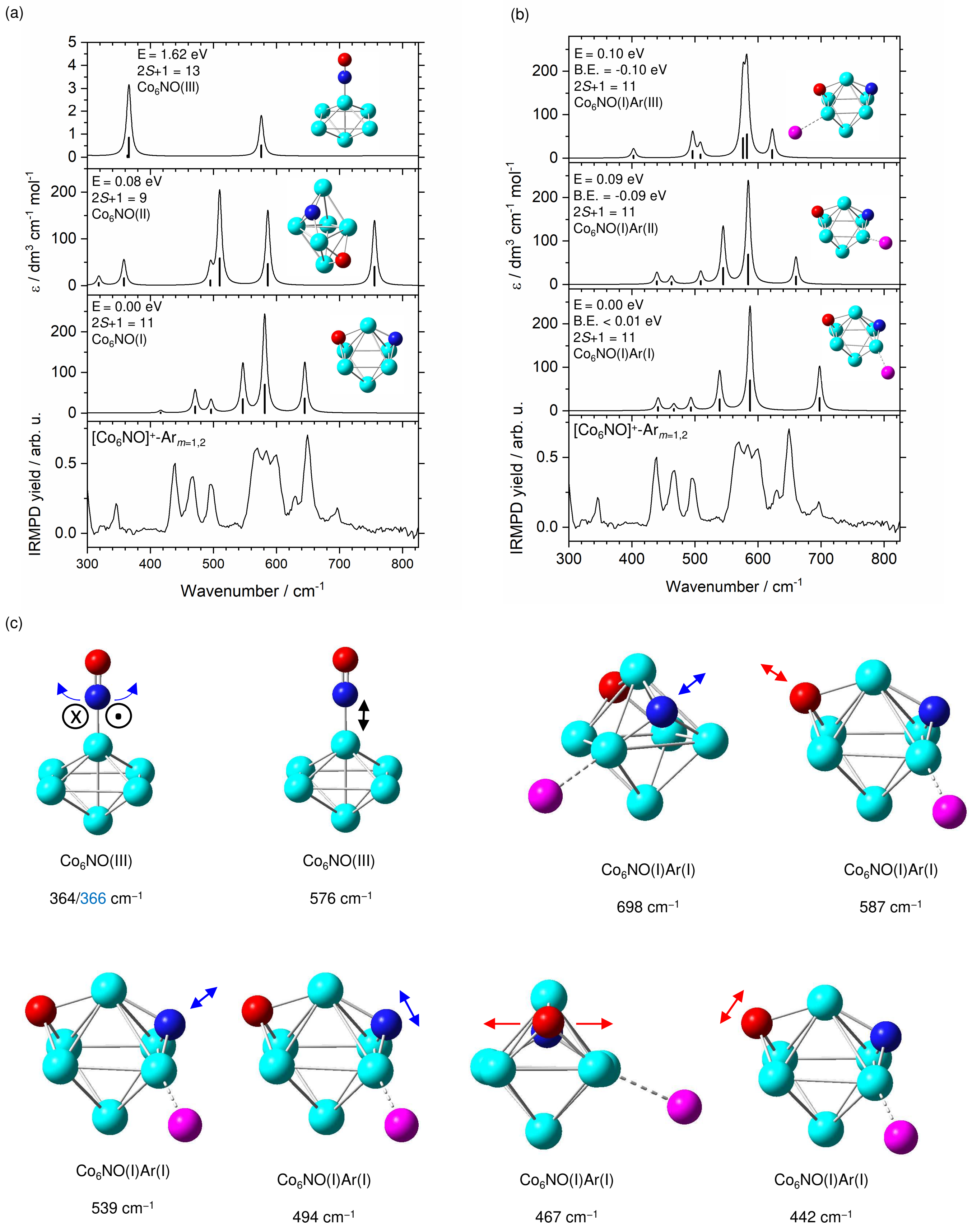

Figure S5: (a) The experimental IRMPD spectrum for $[Co_6NO]^+$-Ar_m compared to spectra calculated for low-lying isomeric forms of $[Co_6NO]^+$ with both molecular and dissociative NO binding motifs. (b) The experimental IRMPD spectrum for $[Co_6NO]^+$-Ar_m compared to spectra calculated for low-lying isomeric forms of $[Co_6NO]^+$-Ar. (c) The frequencies and motions of vibrational modes calculated for the molecularly bound structure, Co_6NO(III), and putatively assigned $[Co_6NO]^+$-Ar structure, Co_6NO(I)Ar(I). Structures are calculated at the B3P86/Def2TZVP level of theory with relative energies (E), spin multiplicities, and binding energies (B.E.) for the Ar tags are also shown.

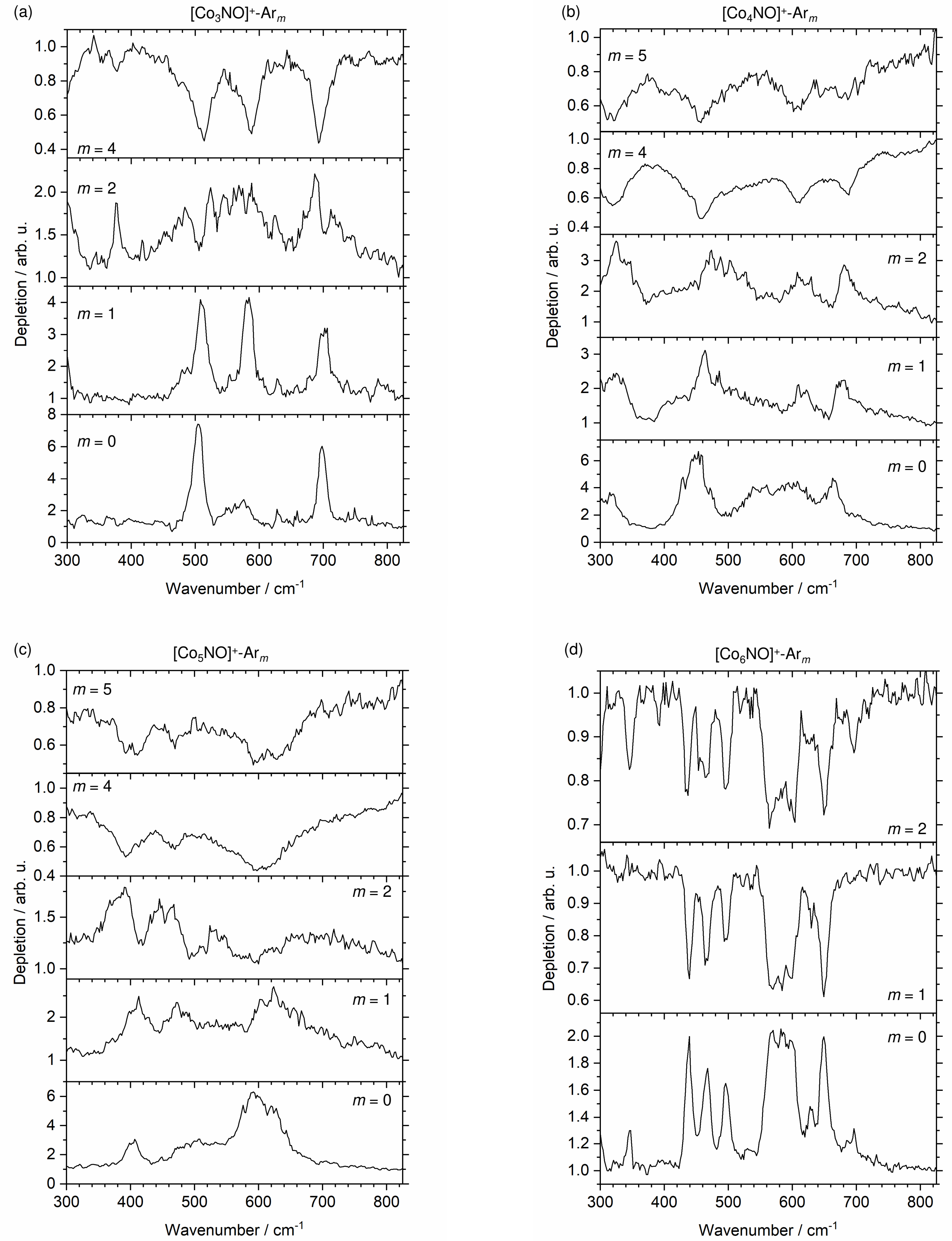

Figure S6: Shown here are the pure ($B(v)/B_0$, as in equation 3 without power correction) depletion spectra of $[Co_nNO]^+$-Ar_m clusters for (a) n = 3, (b) n = 4, (c) n = 5, and (d) n = 6. The number of Ar atoms, m, shown in each panel indicates which mass channel the corresponding depletion spectrum was recorded. Therefore, enhancements in a mass channel $[Co_nNO]^+$-Ar_m mean that this is the spectrum of $[Co_nNO]^+$-Ar_{m+1}, whereas depletion in a mass channel $[Co_nNO]^+$-Ar_m mean that this the spectrum of $[Co_nNO]^+$-Ar_m. For example, in S6 (a) the $[Co_3NO]^+$-Ar_m mass channel for m = 0 exhibits enhancement which must arise from depletion of the channel above where m = 1.

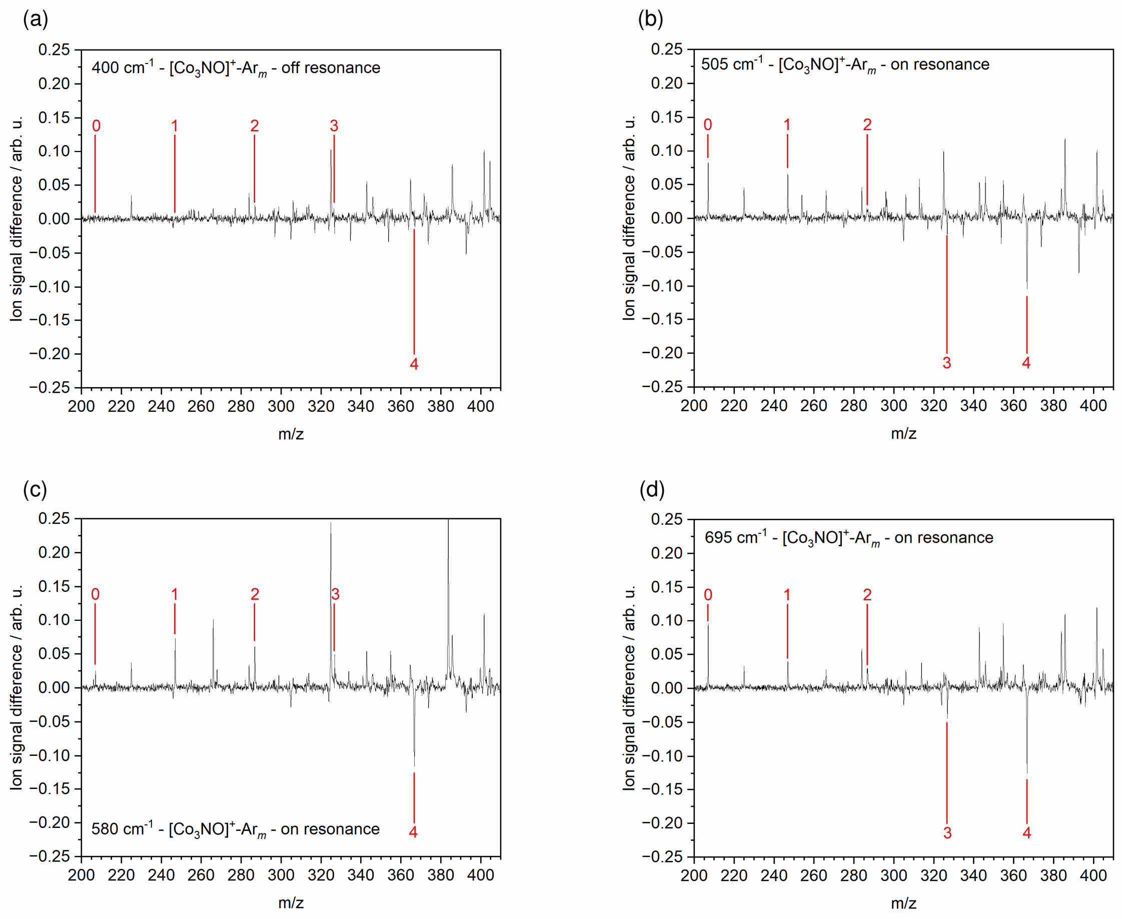

Figure S7: Under the cluster source conditions (backing pressure and argon partial pressure) suitable for singly tagging the larger complexes, the smaller clusters (n = 3–5) bind up to 5 argon atoms. This, combined with the high binding energies of Ar calculated for these sizes, leads to differences observed between the individual $[Co_nNO]^+$-Ar_m depletion spectra (Figure S6), including some mass channels exhibiting enhancement rather than depletion. To be certain that the spectra generated arise from IR-induced loss of Ar rather than undesired ingrowth from depletion of other mass channels, mass difference spectra for $[Co_3NO]^+$-Ar_m are taken at wavenumbers corresponding to resonant modes 505, 580, and 695 cm^{-1} and “off resonance” (400 cm^{-1}) as shown in (a), (b), (c), and (d) respectively. It is clear that depletion of $[Co_3NO]^+$-Ar_4 coincides with enhancement of $[Co_3NO]^+$-$Ar_{m=0\text{-}2}$, thus supporting the spectral assignments made. Similar mass difference spectra are shown in Figures S8 and S9 for the $[Co_nNO]^+$-Ar_m cluster sizes n = 4 and 5, respectively. Note that $[Co_nNO]^+$-Ar_3 is mass degenerate with $[Co_{n+2}O_2]^+$ and was therefore not included in the generation of spectra for $[Co_nNO]^+$-Ar_m.

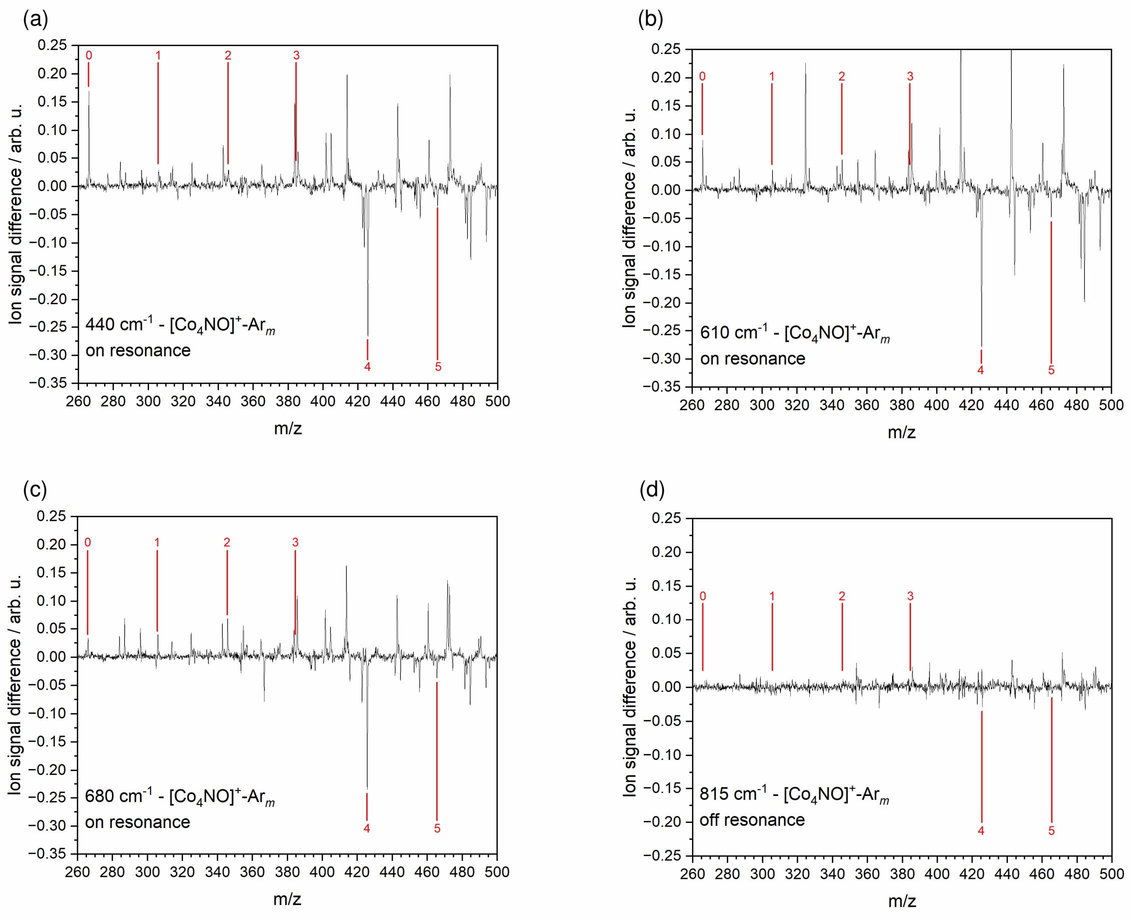

Figure S8: Mass difference spectra for $[Co_4NO]^+$-Ar$_m$ are taken at wavenumbers corresponding to resonant modes 440, 610, and 680 cm^{-1} and “off resonance” (815 cm^{-1}) as shown in (a), (b), (c), and (d) respectively.

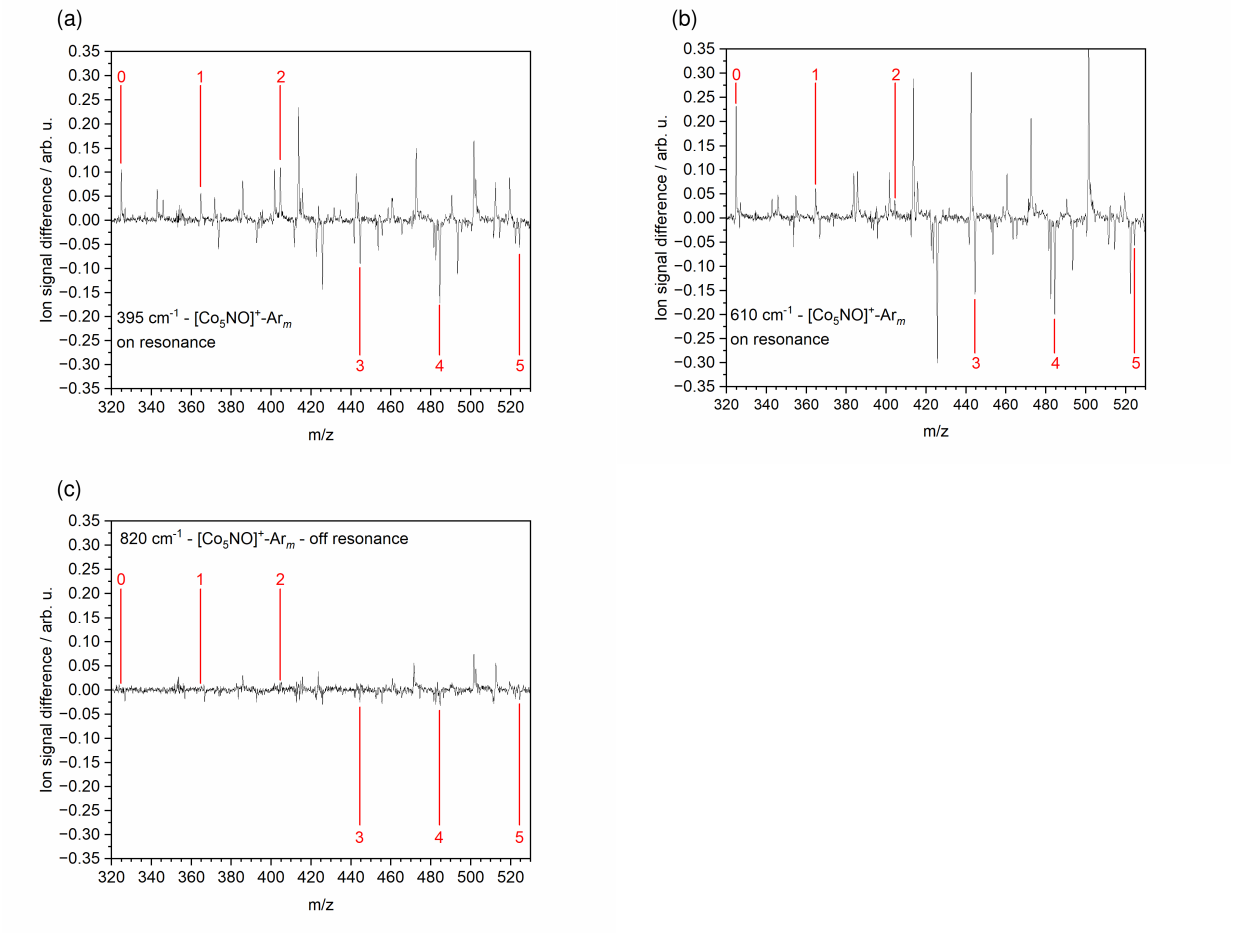

Figure S9: Mass difference spectra for $[Co_5NO]^+$-Ar$_m$ are taken at wavenumbers corresponding to resonant modes 395 and 610 cm^{-1}, and “off resonance” (820 cm^{-1}) as shown in (a), (b), and (c) respectively.

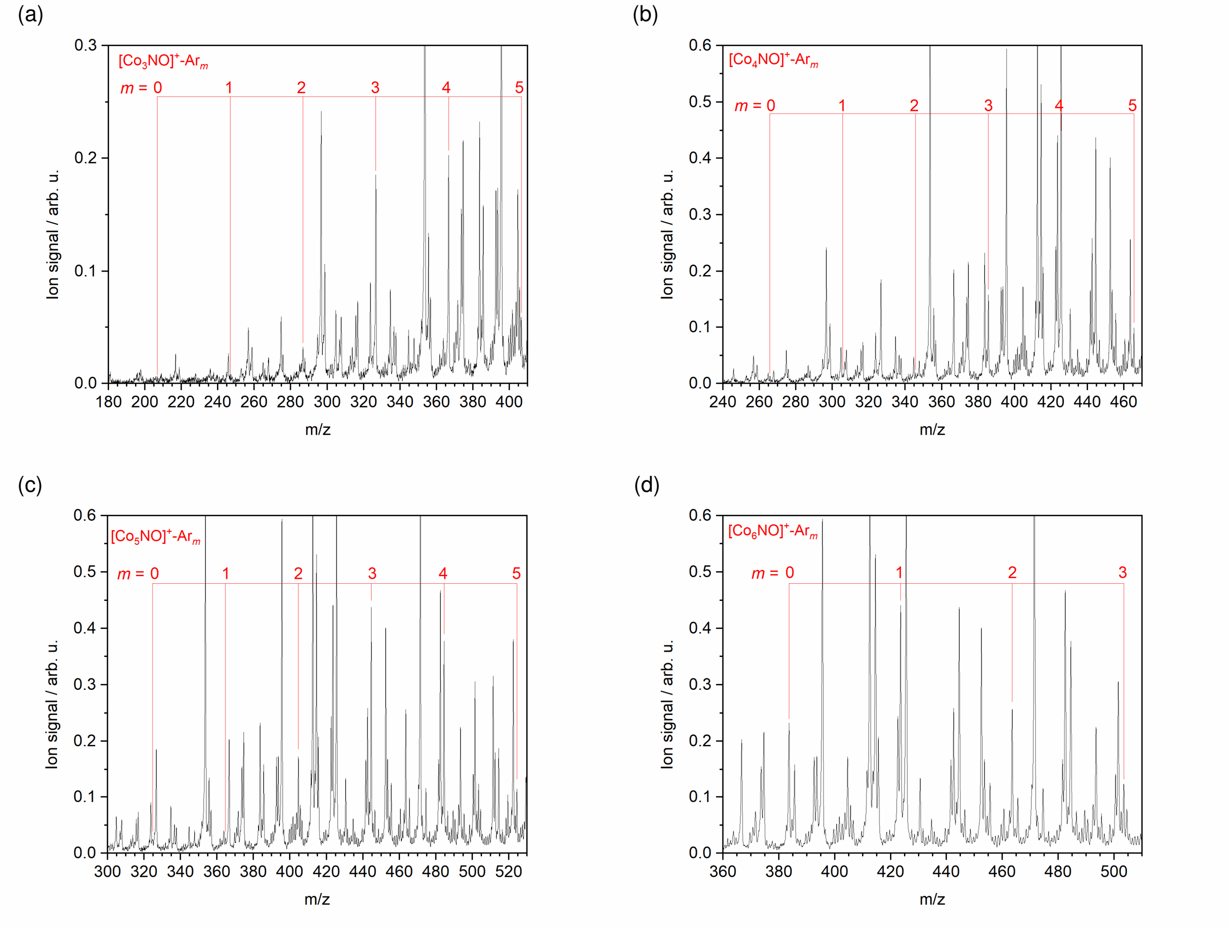

Figure S10: The negative IRMPD yields observed for $[Co_3NO]^+$-$Ar_{m=1\text{-}3}$ (Figure S6, measured as enhancements in the mass channels $[Co_3NO]^+$-$Ar_{m=0\text{-}2}$) can be explained by inspection of the mass spectrum. Figure S10 shows mass spectra highlighting the intensity ratios between successively Ar-tagged $[Co_nNO]^+$ (n = 3–6). This illustrates that, in our source conditions, the smaller (n = 3-5) clusters are more prone to over-clustering with Ar than larger $[Co_nNO]^+$ clusters. For the smaller clusters, upon resonant IR absorption, the mass channel $[Co_nNO]^+$-Ar experiences depletion by loss of Ar as well as ingrowth from $[Co_nNO]^+$-Ar_2, resulting in an overall gain in intensity due to the depletion from the more intense $[Co_nNO]^+$-Ar_2 channel dominating. This changes for the $[Co_nNO]^+$-Ar_4 mass channel as the $[Co_nNO]^+$-Ar_5 channel is less intense. Additionally, the high strength of Ar-binding to the $[Co_{n=3\text{-}5}NO]^+$ clusters may result in kinetic trapping of clusters in the $[Co_{n=3\text{-}5}NO]^+$-$Ar_{m=1\text{-}2}$ mass channels upon loss of Ar. (a), (b), and (c) show the Ar tag branching ratios of $[Co_nNO]^+$-Ar_m clusters for n = 3, 4, and 5 respectively. Such behavior is not exhibited for clusters of $n \geq 6$ as the $[Co_nNO]^+$-$Ar_{m\geq 1}$ mass channel intensity decreases with increasing m, thus depletion would dominate over ingrowth. An example of this branching ratio type is shown in (d) for $[Co_6NO]^+$-$Ar_{m=0\text{-}3}$.

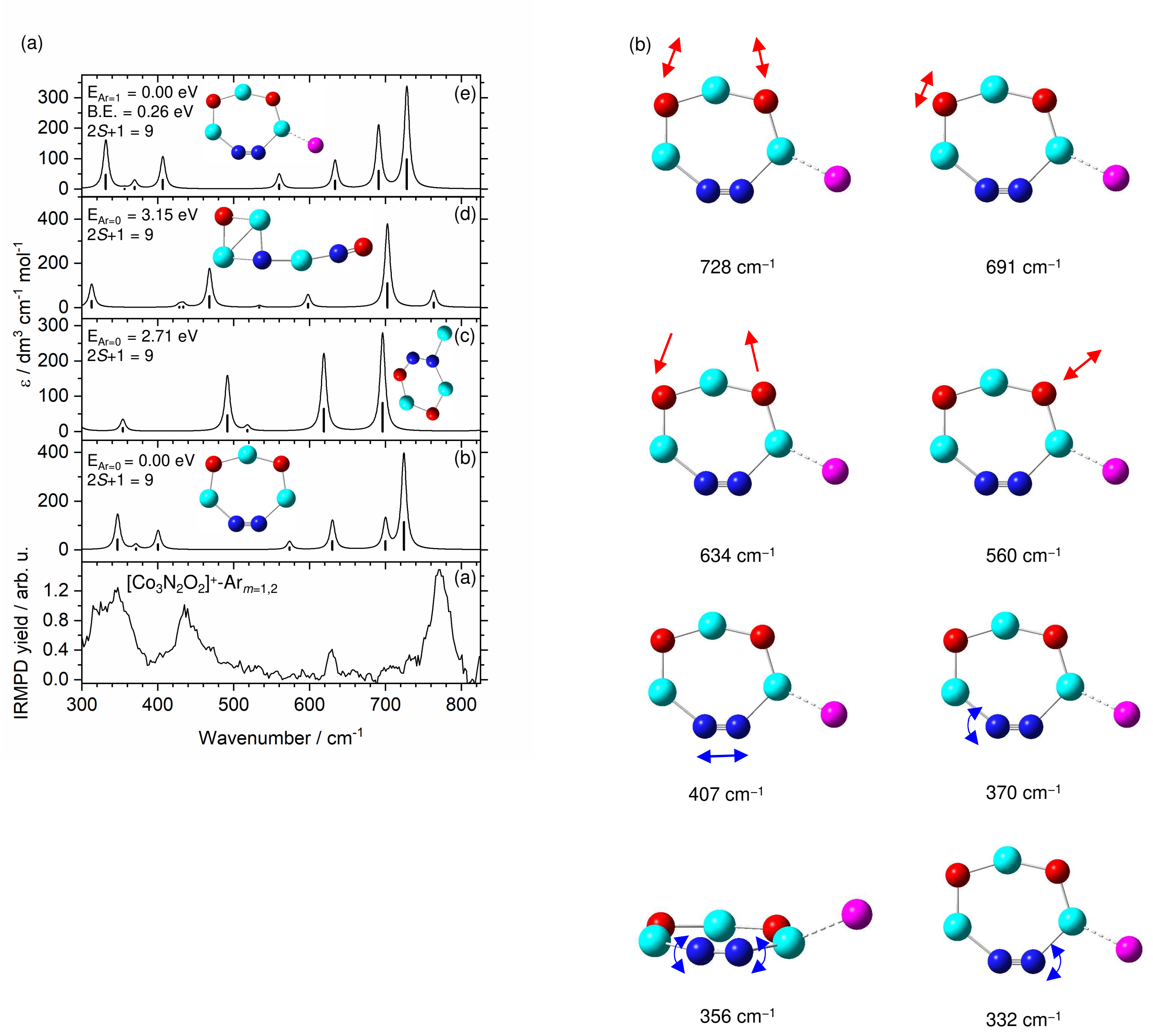

Figure S11: (a) Comparison between the experimental IRPD spectrum of $[Co_3N_2O_2]^+$-Ar_m (m = 1,2) and the harmonic spectra of low-energy isomeric structures of $[Co_3N_2O_2]^+$-Ar_m (m = 0,1) calculated at the B3P86/Def2TZVP level of theory. The energies of the structures ($E_{Ar=m}$) are relative to the energy of the lowest energy isomer calculated for the same number of Ar tags. Calculated binding energies (B.E.) for the Ar tag and spin multiplicities are also shown. (b) Show here are the frequencies and motions of vibrational modes calculated for the putatively assigned $[Co_3N_2O_2]^+$-Ar structure. The strong band at 770 cm^{-1} is assigned to an in-phase dioxide stretch, while the bands at 347 and 435 cm^{-1} are assigned to in-plane N=N rocking and wagging motions.

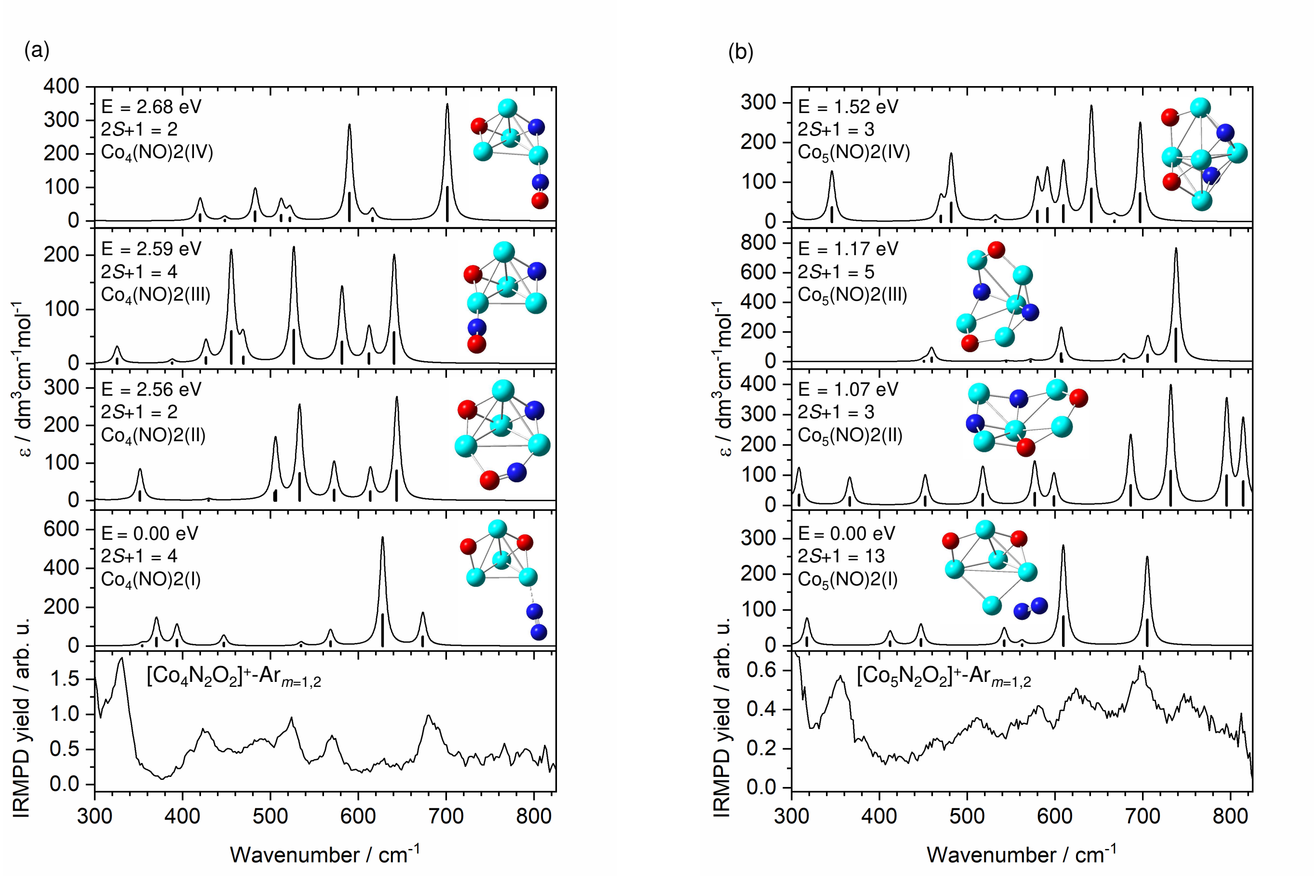

Figure S12: (a) Comparison between the experimental IRPD spectrum of $[Co_4N_2O_2]^+$-Ar_m (m = 1,2) and the harmonic spectra of low-energy isomeric structures of $[Co_5N_2O_2]^+$ calculated at the B3P86/Def2TZVP level of theory. The energies of the structures are relative to the energy of the lowest energy isomer, and their spin multiplicities are also indicated. (b) Comparison between the experimental IRPD spectrum of $[Co_5N_2O_2]^+$-Ar_m (m = 1,2) and the harmonic spectra of low-energy isomeric structures of $[Co_5N_2O_2]^+$ calculated at the B3P86/Def2TZVP level of theory.

The lowest energy $[Co_4N_2O_2]^+$ structure comprises a decorated tetrahedral dioxide cluster, $[Co_4O_2]^+$, with atop-bound N_2 molecule which is consistent with N2 loss in Co_nNO^+ + NO reactions under single collision conditions.[1] However, the exothermicity associated with forming N_2 would almost certainly result in N_2 loss meaning this global minimum structure is unlikely to be observed experimentally. Higher-lying calculated structures (Figure S12 (a)) all show intact NO molecules binding at different sites on the underlying $[Co_4NO]^+$ cluster. However, comparison between experimental and calculated spectra for $[Co_4N_2O_2]^+$ does not reveal a convincing candidate for structural assignment. All the calculated structures lie within a narrow energy range of 0.12 eV, and exhibit multiple bands between 400 and 700 cm^{-1}.

Similarly for $[Co_5N_2O_2]^+$-Ar_m, the lowest energy calculated isomer, $Co_5(NO)_2$(**I**) (Figure S12 (b)), is a dioxide cluster with molecularly bound N2. The higher energy, Co_5(NO)2(**II**–**IV**), however, are more interesting than for n = 4. $Co_5(NO)_2$(**II**) and $Co_5(NO)_2$(**III**) both reflect dissociative adsorption of the second NO molecule which results in breaking open the underlying trigonal bipyramidal Co_5^+ structure. These structures lie significantly (0.35–0.45 eV) lower in energy than the more intuitive isomer, $Co_5(NO)_2$(**IV**), which exhibits dissociative binding of the second NO on $[Co_5NO]^+$ while maintaining the underlying structure. No conclusive assignment is possible but the simulated spectrum of $Co_5(NO)_2$(**II**) matches many of the observed experimental bands.

(1) Anderson, M. L.; Lacz, A.; Drewello, T.; Derrick, P. J.; Woodruff, D. P.; Mackenzie, S. R. The chemistry of nitrogen oxides on small size-selected cobalt clusters, Co_n^+. *J. Chem. Phys.* **2009**, *130* (6), 064305

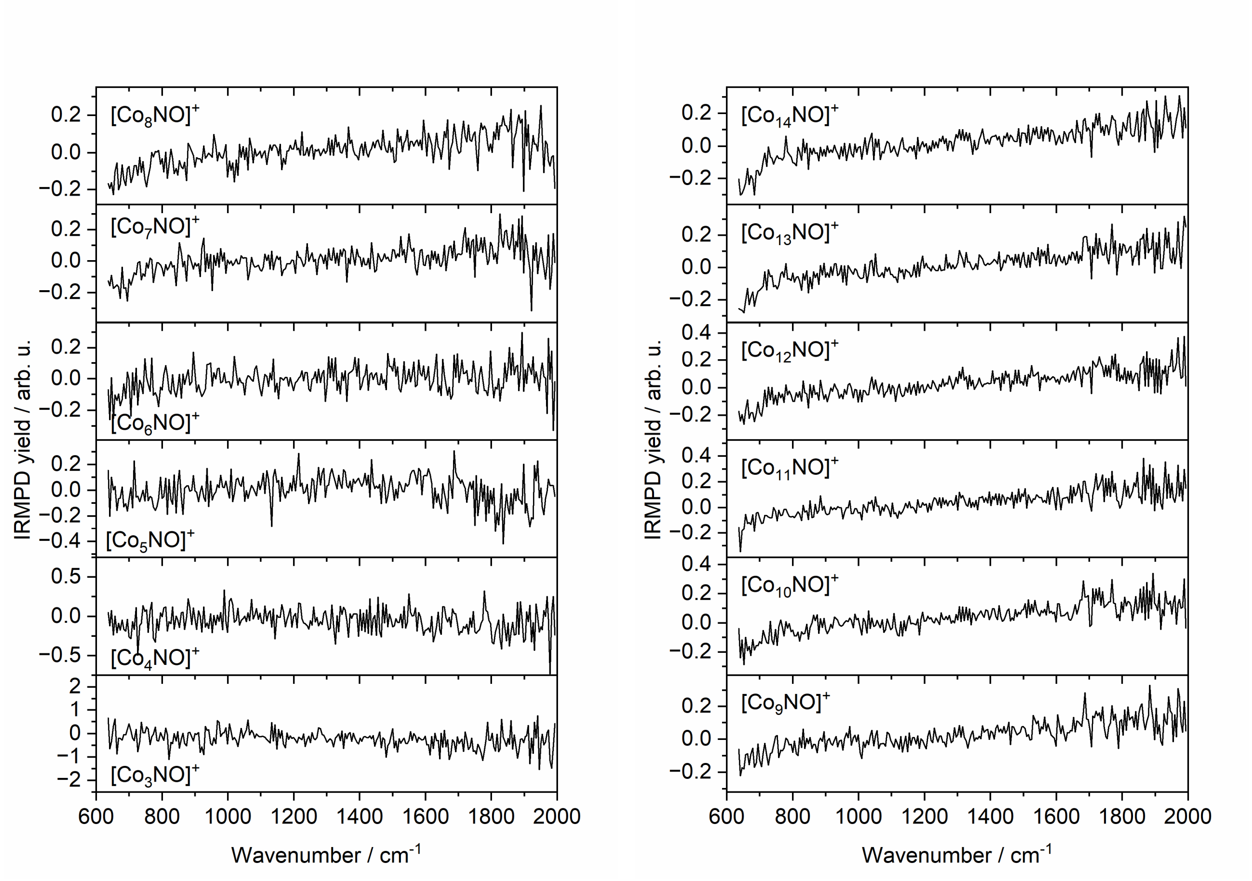

Figure S13: IRMPD spectra of $[Co_nNO]^+$ (n = 3-14) species recorded in the 630–2000 cm^{-1} wavelength range. The enhancements exhibited by $n > 5$ below ~700 cm^{-1} are attributed to depletions of species 18 amu higher in mass which are tentatively assigned to $[Co_nNO]^+$-H_2O clusters.

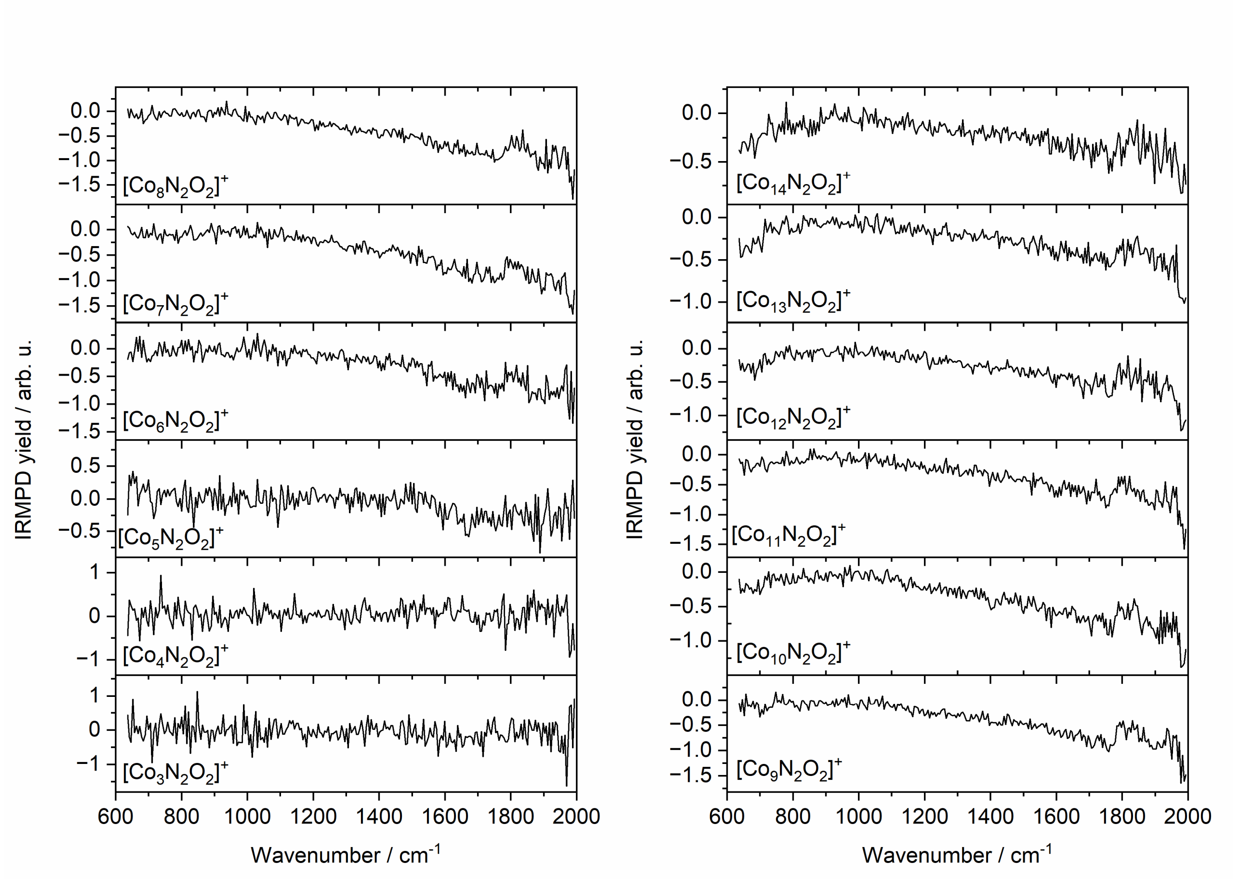

Figure S14: IRMPD spectra of $[Co_nN_2O_2]^+$ (n = 3-14) species recorded in the 630–2000 cm^{-1} wavelength range. The broad enhancements exhibited by $n > 5$ from ~1400–2000 cm^{-1} are attributed to depletions of species 18 amu higher in mass which are tentatively assigned to $[Co_nN_2O_2]^+$-H_2O clusters.

Structure	Atom	x	y	z
$[Co_3NO]^+$ dissociatively bound	Co	1.402685	-0.965489	-0.000018
	Co	-2.131811	-0.274638	0.000035
	Co	0.256712	1.172223	-0.000047
	N	-0.446446	-0.528385	-0.000086
	O	1.985039	0.691511	0.000175
$[Co_3NO]^+$ molecularly bound	Co	-1.288481	-1.195192	-0.000106
	Co	-1.287837	1.195422	-0.000106
	Co	0.833164	-0.000110	0.000384
	N	2.528214	-0.000207	-0.000132
	O	3.670954	-0.000227	-0.000466
$[Co_3NO]^+$-Ar	Co	2.152818	0.965198	0.000653
	Co	-1.413221	0.295433	-0.005639
	Co	0.932914	-1.179771	-0.004585
	N	0.268967	0.513726	-0.003307
	O	2.652182	-0.737016	0.018161
	Ar	-3.792113	0.006491	0.007570
$[Co_3NO]^+$-Ar_3	Co	-0.440465	1.554464	-0.505921
	Co	-2.123955	-0.351437	0.217545
	Co	1.441084	0.067237	-0.059509
	N	-0.439129	-0.082173	0.221420
	O	1.278423	1.716716	-0.815897
	Ar	-4.469141	-0.829108	0.253546
	Ar	2.710047	-1.703638	-1.372928
	Ar	3.046683	-0.103679	1.917722

Table S1: Cartesian coordinates, in Å, of the $[Co_3NO]^+$-Ar_m (m = 0, 1, 3) structures displayed in Figure 3.

Structure	Atom	x	y	z
I1	Co	-1.329222	1.178035	0.000611
	Co	-1.326661	-1.179510	0.000607
	Co	0.888438	0.001471	-0.002121
	N	2.573138	0.000881	-0.000501
	O	3.713633	-0.000759	0.003486
I2	Co	1.811822	0.029753	0.000068
	Co	-0.337681	1.238389	-0.000056
	Co	-0.295648	-1.242028	-0.000103
	N	-1.923763	-0.654448	0.000356
	O	-2.294121	0.484507	-0.000006
I3	Co	1.635412	-0.729711	0.076043
	Co	0.350571	1.444115	-0.073423
	Co	-1.899538	-0.322663	0.066194
	N	-0.097907	-0.232757	0.461243
	O	-0.206082	-1.118460	-0.635834
I4	Co	1.910343	-0.461679	0.124157
	Co	-1.813517	-0.414280	0.184447
	Co	-0.003721	1.361684	-0.109440
	N	0.088070	-0.437179	0.152707
	O	-0.391291	-1.256792	-0.805795
I5	Co	-1.417203	-0.952068	-0.004460
	Co	-0.204987	1.160574	-0.054433
	Co	2.080688	-0.298677	0.057718
	N	0.414382	-0.527176	-0.224564
	O	-1.910017	0.765608	0.200458
GM	Co	1.402685	-0.965489	-0.000018
	Co	-2.131811	-0.274638	0.000035
	Co	0.256712	1.172223	-0.000047
	N	-0.446446	-0.528385	-0.000086
	O	1.985039	0.691511	0.000175
TS1	Co	-1.824150	-0.195564	0.006796
	Co	0.069836	1.321341	-0.007208
	Co	0.457383	-1.123453	-0.005154
	N	2.080821	-0.573970	-0.008344
	O	2.556425	0.494381	0.026085
TS2	Co	1.497794	-0.517343	-0.153729
	Co	0.150043	1.437228	0.034123
	Co	-1.598726	-0.329139	-0.164193
	N	-0.152191	-0.444228	0.906773
	O	-0.032583	-1.605066	0.164396
TS3	Co	2.044114	0.019627	-0.065900
	Co	-1.136204	-1.194496	0.026310
	Co	-1.212674	1.148533	-0.084007
	N	0.588632	-0.926454	-0.038080
	O	0.513525	0.899530	0.450458

Table S2: Cartesian coordinates, in Å, of the $[Co_3NO]^+$ structures displayed in the reaction pathway in Figure 4. This includes intermediate structures, transition state structures, and the final global minimum structure, GM.

Structure	Atom	x	y	z
Co_4NO(II)Ar	Co	-1.194084	-0.486226	-0.223822
	Co	1.321723	0.176310	-1.253040
	Co	0.364334	1.423219	0.526354
	Co	1.195081	-1.049730	0.912393
	N	0.284663	-1.280005	-0.659976
	O	2.003302	0.587990	0.545341
	Ar	-3.531640	0.141091	0.071454
Co_5NO(II)Ar(I)	Co	-2.590199	0.528162	-0.228567
	Co	-0.651226	-0.776061	-1.220191
	Co	-0.124091	1.341126	-0.021391
	Co	1.746674	-0.489541	-0.000957
	Co	-0.687753	-0.803888	1.214173
	N	-1.472084	0.838053	1.080078
	O	0.492779	-1.744333	0.053195
	Ar	3.813356	0.749653	-0.058272
Co_6NO(I)Ar(I)	Co	0.904246	-1.811782	0.228602
	Co	2.130260	0.179087	-0.574961
	Co	0.056994	-0.463398	-1.630154
	Co	-1.600826	-0.256017	0.405938
	Co	0.036175	1.628640	-0.415878
	Co	0.747769	0.414176	1.621102
	N	-0.343495	-1.033108	1.284542
	O	1.656211	1.624590	0.480792
	Ar	-4.014441	0.143666	-0.165205

Table S3: Cartesian coordinates, in Å, of the $[Co_nNO]^+$-Ar (n = 4, 5, 6) structures displayed in Figure 5.

Structure	Atom	x	y	z
$[Co_3N_2O_2]^+$ (E = 0.00 eV)	Co	1.934302	-0.621140	0.000631
	Co	-1.934738	-0.620518	0.000203
	Co	0.000430	1.567108	-0.000745
	N	0.565411	-1.893058	-0.001217
	O	1.694961	1.106627	0.000050
	N	-0.566307	-1.892519	-0.001076
	O	-1.694156	1.107360	0.001655
$[Co_3N_2O_2]^+$ (E = 2.71 eV)	Co	-0.379265	-1.285559	0.005366
	Co	2.864325	0.078001	0.001797
	Co	-2.100652	0.657317	0.013400
	N	0.953253	0.121828	-0.000805
	O	-2.130193	-1.116423	-0.037654
	N	0.680138	1.334500	-0.012132
	O	-0.596402	1.699199	-0.020425
$[Co_3N_2O_2]^+$ (E = 3.15 eV)	Co	-2.036090	-0.832287	-0.101766
	Co	1.414764	-0.512775	0.070584
	Co	-0.626394	1.146838	0.162575
	N	-0.319854	-0.560315	0.543349
	O	-2.222703	0.921050	-0.485356
	N	3.015398	-0.078919	-0.158856
	O	4.075158	0.307285	-0.294526
$[Co_3N_2O_2]^+$-Ar	Co	2.235174	-1.113835	-0.112140
	Co	-1.470554	0.069178	0.351378
	Co	1.159694	1.495210	-0.042025
	N	0.571016	-1.861283	0.153994
	O	2.638783	0.576275	-0.269609
	N	-0.515900	-1.581059	0.293589
	O	-0.578976	1.568292	0.157201
	Ar	-3.823374	-0.290283	-0.419921

Table S4: Cartesian coordinates, in Å, of the $[Co_3N_2O_2]^+$-Ar_m (m = 0, 1) structures displayed in Figure 7.

Structure	Mode	Frequency (cm^{-1})	Intensity (km mol^{-1})
$[Co_3NO]^+$ dissociatively bound	ω_1	75.5453	0.9483
	ω_2	77.3144	4.4183
	ω_3	219.1762	62.574
	ω_4	231.3663	3.9543
	ω_5	270.1596	7.4079
	ω_6	499.2268	71.1769
	ω_7	534.0347	55.4591
	ω_8	706.0845	80.4003
	ω_9	826.2677	13.9695
$[Co_3NO]^+$ molecularly bound	ω_1	22.4565	0.0027
	ω_2	31.5489	0.0135
	ω_3	131.2662	1.4738
	ω_4	154.6234	2.5989
	ω_5	227.874	0.8081
	ω_6	249.5091	0.4072
	ω_7	283.2144	0.9668
	ω_8	521.8059	19.0865
	ω_9	1967.584	1108.822
$[Co_3NO]^+$-Ar	ω_1	34.8867	0.6707
	ω_2	37.6285	0.6362
	ω_3	103.9743	3.6104
	ω_4	115.1297	2.1553
	ω_5	147.4053	3.3518
	ω_6	221.4643	38.4239
	ω_7	233.5501	35.0821
	ω_8	278.0898	1.6511
	ω_9	466.8295	98.5609
	ω_{10}	555.4885	52.0633
	ω_{11}	690.8828	77.4757
	ω_{12}	860.4698	47.5442
$[Co_3NO]^+$-Ar_3	ω_1	15.462	0.1318
	ω_2	29.7264	0.0942
	ω_3	39.365	0.6415
	ω_4	42.4529	0.029
	ω_5	48.4758	0.0731
	ω_6	55.5902	0.7081
	ω_7	82.6792	0.4945
	ω_8	118.2553	1.6734
	ω_9	124.8221	3.2779
	ω_{10}	134.5309	1.8111
	ω_{11}	148.7209	10.7571
	ω_{12}	216.9914	37.7366
	ω_{13}	242.9979	21.3729
	ω_{14}	288.4035	15.1628
	ω_{15}	401.7518	67.8075
	ω_{16}	535.564	27.8215
	ω_{17}	699.5238	100.5158
	ω_{18}	771.3622	76.033

Table S5: Calculated harmonic frequencies of the $[Co_3NO]^+$-Ar_m (m = 0, 1, 3) structures displayed in Figure 3.

Structure	Mode	Frequency (cm^{-1})	Intensity (km mol^{-1})
I1	ω_1	15.5126	0.0626
	ω_2	32.414	0.0086
	ω_3	142.1347	2.2509
	ω_4	148.8053	0.5696
	ω_5	208.6315	0.723
	ω_6	224.4458	1.0252
	ω_7	258.5595	1.3157
	ω_8	531.0174	19.6688
	ω_9	1988.834	928.1884
I2	ω_1	58.9938	1.5066
	ω_2	149.6293	3.0007
	ω_3	164.8314	2.8349
	ω_4	177.6201	3.8209
	ω_5	192.2617	0.0689
	ω_6	236.6092	0.8133
	ω_7	343.2174	2.7705
	ω_8	539.7098	9.9303
	ω_9	1594.001	451.8292
I3	ω_1	32.944	4.2174
	ω_2	105.1962	7.7703
	ω_3	130.802	6.246
	ω_4	153.8323	5.2661
	ω_5	259.2347	10.3984
	ω_6	301.5278	15.3127
	ω_7	500.2695	10.6758
	ω_8	659.9033	5.8297
	ω_9	842.3671	80.658
I4	ω_1	55.6455	11.9042
	ω_2	77.7342	1.3948
	ω_3	119.5963	8.0734
	ω_4	142.9577	4.1295
	ω_5	213.5449	3.1519
	ω_6	288.7412	8.3141
	ω_7	500.3091	5.8753
	ω_8	618.6293	27.7588
	ω_9	992.0596	132.6149
I5	ω_1	46.0992	0.8367
	ω_2	95.671	6.6509
	ω_3	186.888	27.9752
	ω_4	235.4847	18.8056
	ω_5	285.7329	17.5462
	ω_6	395.3124	52.5864
	ω_7	532.617	20.3025
	ω_8	695.904	91.7188
	ω_9	754.6914	61.1131

Table S6: Calculated harmonic frequencies of the $[Co_3NO]^+$ structures displayed in the reaction pathway in Figure 4. This includes intermediate structures, transition state structures, and the final global minimum structure, GM.

Structure	Mode	Frequency (cm^{-1})	Intensity (km mol^{-1})
GM	ω_1	75.5453	0.9483
	ω_2	77.3144	4.4183
	ω_3	219.1762	62.574
	ω_4	231.3663	3.9543
	ω_5	270.1596	7.4079
	ω_6	499.2268	71.1769
	ω_7	534.0347	55.4591
	ω_8	706.0845	80.4003
	ω_9	826.2677	13.9695
TS1	ω_1	-104.639	0.4982
	ω_2	49.4333	0.8109
	ω_3	136.1277	5.1726
	ω_4	160.4076	2.4136
	ω_5	173.34	0.4792
	ω_6	233.0819	0.408
	ω_7	273.9287	0.0247
	ω_8	537.6145	11.3646
	ω_9	1746.879	566.9702
TS2	ω_1	-192.537	6.0544
	ω_2	58.4879	4.6921
	ω_3	173.5121	5.5547
	ω_4	177.2827	7.6349
	ω_5	241.2194	2.6102
	ω_6	348.9629	5.3447
	ω_7	410.6692	17.0947
	ω_8	546.438	22.7255
	ω_9	868.0498	205.4715
TS3	ω_1	-428.192	54.2836
	ω_2	60.317	17.7433
	ω_3	123.9654	6.3963
	ω_4	171.178	8.4151
	ω_5	239.3454	2.0733
	ω_6	318.5554	17.1824
	ω_7	427.6123	19.6432
	ω_8	610.8257	19.1358
	ω_9	713.3596	30.2186

Table S6: (continued.) Calculated harmonic frequencies of the $[Co_3NO]^+$ structures displayed in the reaction pathway in Figure 4. This includes intermediate structures, transition state structures, and the final global minimum structure, GM.

Structure	Mode	Frequency (cm^{-1})	Intensity (km mol^{-1})
Co_4NO(II)Ar	ω_1	37.0526	0.1095
	ω_2	48.8323	0.2121
	ω_3	95.9362	5.3711
	ω_4	110.519	4.4942
	ω_5	125.7167	2.423
	ω_6	136.0168	1.8989
	ω_7	181.0723	5.1283
	ω_8	191.6071	3.0634
	ω_9	253.8416	7.2594
	ω_{10}	281.89	2.6504
	ω_{11}	441.9837	3.5968
	ω_{12}	457.6134	73.0487
	ω_{13}	573.2317	67.7395
	ω_{14}	606.9568	21.6259
	ω_{15}	657.93	65.4884
Co_5NO(II)Ar(I)	ω_1	30.4529	0.334
	ω_2	44.1532	0.1241
	ω_3	67.0566	0.5098
	ω_4	86.722	0.8074
	ω_5	102.5031	3.5381
	ω_6	108.5738	3.3592
	ω_7	132.9449	1.0657
	ω_8	145.6857	6.2391
	ω_9	160.1187	0.5396
	ω_{10}	168.9867	4.5025
	ω_{11}	177.9747	7.3997
	ω_{12}	236.5717	2.6332
	ω_{13}	333.3305	16.1476
	ω_{14}	481.5671	10.3164
	ω_{15}	495.3293	33.8218
	ω_{16}	585.1088	28.6196
	ω_{17}	610.8061	23.2632
	ω_{18}	623.8137	45.4982
Co_6NO(I)Ar(I)	ω_1	23.4914	0.1022
	ω_2	38.0255	0.1436
	ω_3	55.1893	2.3024
	ω_4	90.9668	2.6209
	ω_5	110.086	1.0437
	ω_6	111.613	2.0721
	ω_7	120.9432	1.74
	ω_8	127.5157	1.2471
	ω_9	139.6493	2.9906
	ω_{10}	149.9702	9.4958
	ω_{11}	167.1422	2.5563
	ω_{12}	177.8283	10.2855
	ω_{13}	190.9423	10.4559
	ω_{14}	202.749	3.2442
	ω_{15}	231.9324	1.7201
	ω_{16}	441.5432	8.525
	ω_{17}	466.5024	4.2809
	ω_{18}	493.5584	8.4845
	ω_{19}	539.16	26.3336
	ω_{20}	587.286	69.6579
	ω_{21}	697.6022	29.6379

Table S7: Calculated harmonic frequencies of the $[Co_nNO]^+$-Ar (n = 4, 5, 6) structures displayed in Figure 5.

Structure	Mode	Frequency (cm^{-1})	Intensity (km mol^{-1})
$[Co_3N_2O_2]^+$ (E = 0.00 eV)	ω_1	77.68	31.8306
	ω_2	79.5866	19.8992
	ω_3	113.86	0.0005
	ω_4	115.8894	6.7138
	ω_5	149.6069	44.1405
	ω_6	177.865	0.4813
	ω_7	347.1685	42.238
	ω_8	359.0489	0.0017
	ω_9	371.3583	5.6082
	ω_{10}	400.4321	22.7714
	ω_{11}	573.6151	9.8305
	ω_{12}	630.0667	35.2744
	ω_{13}	699.9102	35.6269
	ω_{14}	724.1506	114.3697
	ω_{15}	2057.076	330.9506
$[Co_3N_2O_2]^+$ (E = 2.71 eV)	ω_1	48.7239	32.2264
	ω_2	57.1186	6.8668
	ω_3	73.7363	3.1587
	ω_4	141.156	3.364
	ω_5	170.3706	4.6011
	ω_6	194.8585	6.6253
	ω_7	288.1256	33.6584
	ω_8	354.0678	9.9109
	ω_9	491.7675	45.8943
	ω_{10}	518.177	4.2677
	ω_{11}	618.7674	63.9554
	ω_{12}	696.1565	80.7779
	ω_{13}	842.2095	14.3201
	ω_{14}	979.679	241.2908
	ω_{15}	1475.699	90.5426
$[Co_3N_2O_2]^+$ (E = 3.15 eV)	ω_1	25.3361	0.3729
	ω_2	40.6448	0.1459
	ω_3	84.6537	0.9688
	ω_4	125.7272	0.7745
	ω_5	171.5041	23.1761
	ω_6	248.1787	16.0264
	ω_7	312.9889	30.5235
	ω_8	428.5297	3.9715
	ω_9	433.6779	4.9579
	ω_{10}	468.1974	51.2729
	ω_{11}	533.6265	2.6325
	ω_{12}	597.8933	17.064
	ω_{13}	702.4832	109.5976
	ω_{14}	763.3625	22.1365
	ω_{15}	2008.888	1311.956

Table S8: Calculated harmonic frequencies of the $[Co_3N_2O_2]^+$-Ar_m (m = 0, 1) structures displayed in Figure 7.

Structure	Mode	Frequency (cm^{-1})	Intensity (km mol^{-1})
$[Co_3N_2O_2]^+$-Ar	ω_1	28.9423	0.0219
	ω_2	40.5793	0.3638
	ω_3	78.6259	24.3782
	ω_4	96.2997	9.2002
	ω_5	113.4507	6.1401
	ω_6	118.7551	1.268
	ω_7	132.3962	15.2963
	ω_8	171.042	34.3936
	ω_9	196.7855	16.8342
	ω_{10}	331.6429	46.6322
	ω_{11}	356.3048	0.7025
	ω_{12}	369.5827	7.7695
	ω_{13}	406.6683	30.6816
	ω_{14}	560.0338	14.3673
	ω_{15}	633.5389	27.3463
	ω_{16}	690.8212	59.996
	ω_{17}	728.0369	97.4956
	ω_{18}	2062.321	312.4949

Table S8: (continued.) Calculated harmonic frequencies of the $[Co_3N_2O_2]^+$-Ar_m (m = 0, 1) structures displayed in Figure 7.
